# Annexin 2A sustains glioblastoma cell dissemination and proliferation

**DOI:** 10.18632/oncotarget.10565

**Published:** 2016-07-13

**Authors:** Francesca Maule, Silvia Bresolin, Elena Rampazzo, Daniele Boso, Alessandro Della Puppa, Giovanni Esposito, Elena Porcù, Stefania Mitola, Giuseppe Lombardi, Benedetta Accordi, Manuela Tumino, Giuseppe Basso, Luca Persano

**Affiliations:** ^1^ Department of Woman and Child Health, University of Padova, Padova, IT; ^2^ Istituto di Ricerca Pediatrica (IRP) – Città della Speranza, Padova, IT; ^3^ Neurosurgery Unit, University-Hospital of Padova, Padova, IT; ^4^ Istituto Oncologico Veneto, IRCCS, Padova, IT; ^5^ Experimental Oncology and Immunology Unit, Department of Molecular and Translational Medicine, University of Brescia, Brescia, IT; ^6^ Department of Clinical and Experimental Oncology, Medical Oncology 1, Istituto Oncologico Veneto, IRCCS, Padova, IT; ^7^ Clinic of Pediatric Oncohematology, University-Hospital of Padova, Padova, IT

**Keywords:** annexin 2A, glioblastoma, invasion, cell cycle, cytoskeletal remodeling

## Abstract

Glioblastoma (GBM) is the most devastating tumor of the brain, characterized by an almost inevitable tendency to recur after intensive treatments and a fatal prognosis. Indeed, despite recent technical improvements in GBM surgery, the complete eradication of cancer cell disseminated outside the tumor mass still remains a crucial issue for glioma patients management. In this context, Annexin 2A (ANXA2) is a phospholipid-binding protein expressed in a variety of cell types, whose expression has been recently associated with cell dissemination and metastasis in many cancer types, thus making ANXA2 an attractive putative regulator of cell invasion also in GBM.

Here we show that ANXA2 is over-expressed in GBM and positively correlates with tumor aggressiveness and patient survival. In particular, we associate the expression of ANXA2 to a mesenchymal and metastatic phenotype of GBM tumors. Moreover, we functionally characterized the effects exerted by ANXA2 inhibition in primary GBM cultures, demonstrating its ability to sustain cell migration, matrix invasion, cytoskeletal remodeling and proliferation. Finally, we were able to generate an ANXA2-dependent gene signature with a significant prognostic potential in different cohorts of solid tumor patients, including GBM.

In conclusion, we demonstrate that ANXA2 acts at multiple levels in determining the disseminating and aggressive behaviour of GBM cells, thus proving its potential as a possible target and strong prognostic factor in the future management of GBM patients.

## INTRODUCTION

Glioblastoma multiforme (GBM) is the highest grade glioma (grade IV), considered as the most aggressive primary brain tumor in the adult [1]. GBM is characterized by rapid growth and an extremely invasive phenotype which results in extensive and diffuse infiltration into the surrounding normal brain tissues [2]. Despite decades of treatment optimizations, GBM patients display a median survival of only 17 months [3, 4]. Indeed, failure of multimodal treatments, which include surgery, radio- and adjuvant chemo-therapy, has often been associated to: i) the intrinsic highly infiltrative phenotype of GBM cells [5, 6]; ii) the presence of cancer cells endowed with stem-like features, which have been reported to be resistant to standard chemotherapies [7–9]. In this context, the introduction of 5-ALA in order to improve the identification and surgical eradication of disseminated GBM cells, significantly increased patient survival [10, 11]. However, complete resection could be challenging to accomplish and fully achieved in only a fraction of patients, due to the tumor spreading into eloquent areas [12, 13]. These considerations make particularly relevant the identification and potential targeting of the molecular mechanisms sustaining GBM cell heterogeneity and dissemination.

The mechanisms underlying migration and invasion of GBM cells are complex and involve a sequence of events which includes: i) adhesion of tumor cells to the extracellular matrix (ECM); ii) remodeling and degradation of ECM in order to create a “permissive” extracellular space; iii) invasion of cells into the modified tissue [2]. Remodeling and degradation of the ECM depends on both secretion of altered matrix components and proteolytic cleavage of the existing matrix by proteases, whose expression and activity is tightly regulated [14]. In addition, invasion of GBM cells into this permissive microenvironment can be stimulated by multiple factors that are either secreted by tumor cells themselves or by the surrounding stroma [15]. For all these reasons, any imbalance in the expression of proteases, receptors and soluble factors could dramatically impact the complex process of cancer cell invasion.

Annexin 2A (also called annexin II, ANXA2, calpactin I or lipocortin II), is a calcium-binding cytoskeletal protein expressed on the surface of endothelial cells, macrophages, mononuclear cells and various types of cancer cells [16]. Annexin A2 binds to plasminogen together with tissue plasminogen activator (t-PA) on the cell surface, thus facilitating the conversion of plasminogen into plasmin [17]. Indeed, plasmin is a serine protease which plays a key role in the activation of metalloproteinases and degradation of ECM, an essential step for metastatic cancer progression. Due to this reported function, several studies suggested ANXA2 as an essential regulator of cancer cell adhesion and invasion, but also proliferation [18–20]. Indeed, increased expression of ANXA2 and its positive correlation with cell migration and invasion have been described in several types of cancers including colorectal, pancreatic, breast and renal cancer, gastric carcinoma and vascular tumors [21]. In addition to the effects on adhesion and invasion, ANXA2 also been demonstrated to play an important role in regulating cytoskeleton structures and remodeling of actin fibers, which are both essential steps of a functional cell migration process [22, 23].

In this study, we evaluated ANXA2 protein expression in a cohort of glioma patients, finding a strong correlation with tumor aggressiveness and patient survival. Moreover, by modulating its activity, we generated an ANXA2-dependent gene expression profile strictly correlated to the regulation of fundamental cellular features such as migration, invasion, cytoskeletal remodeling and cell cycle, which have all been examined *in vitro* and *in vivo* in primary human GBM cells. Finally, we created an ANXA2-dependent gene signature able to stratify GBM patients for survival.

## RESULTS

### ANXA2 expression correlates with glioma grade and patient outcome

**Figure 1:**
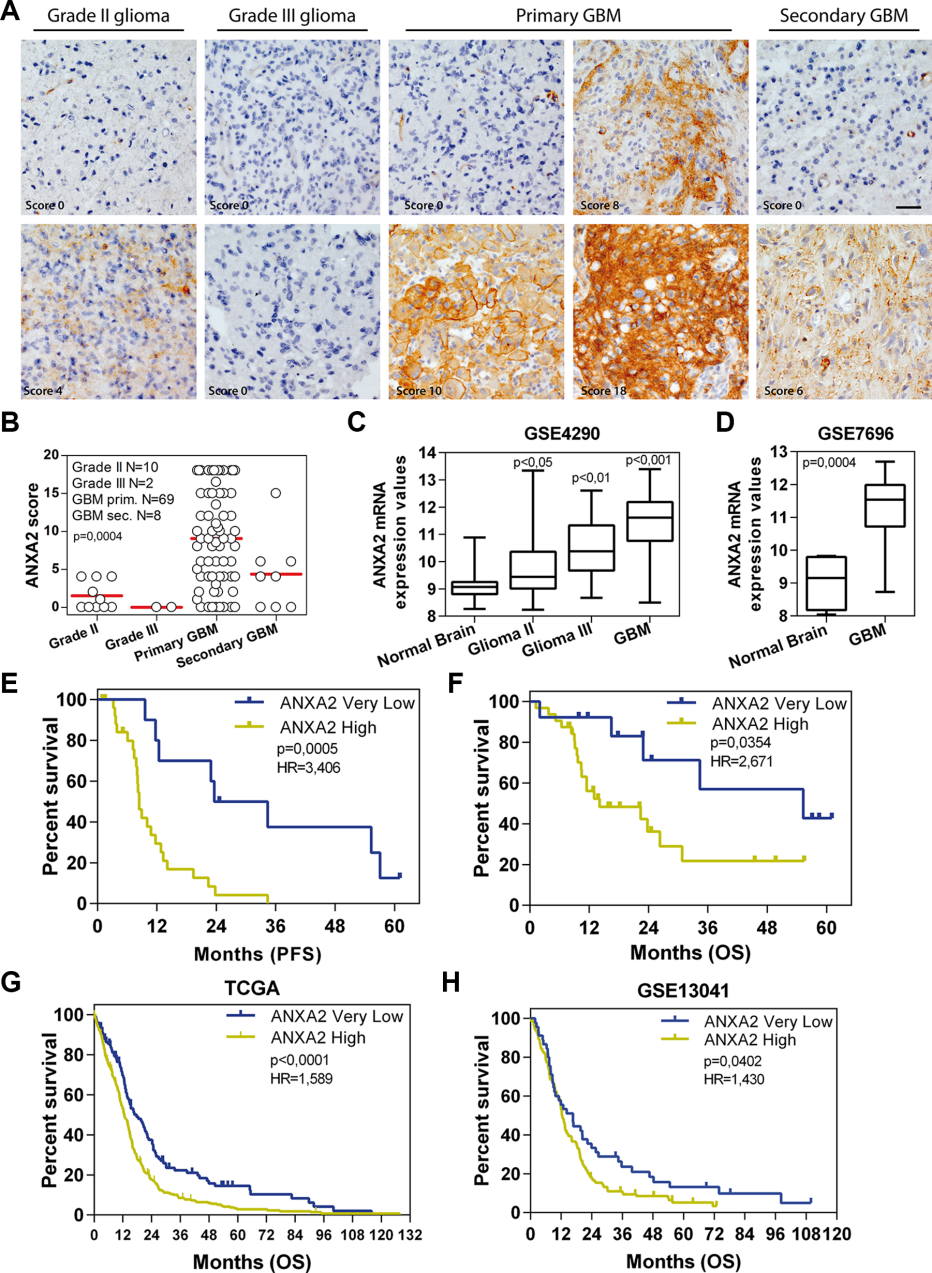
ANXA2 is over-expressed in GBM and positively correlates with bad prognosis (**A**) Representative ANXA2 IHC staining performed on grade II, III and IV gliomas and secondary GBMs. Original magnification 20x; bar:50 μm. (**B**) ANXA2 protein expression levels represented as IHC scores in 10 grade II gliomas, 2 grade III gliomas, 69 GBM and 8 secondary GBM samples. (**C** and **D**) Box plots showing ANXA2 gene expression in samples retrieved from GSE4290 and GSE7696 datasets respectively. *p* values have been calculated relative to Normal Brain samples. (**E** and **F**) Kaplan Meier curves showing the impact of ANXA2 IHC score on GBM patient outcome in terms of progression-free (PFS) (E) and overall survival (OS) (F). (**G** and **H**) Validation of prognostic potential of ANXA2 mRNA expression in TCGA (G; *N* = 519 patients) and GSE13041 (H; *N* = 191) datasets.

To evaluate the impact of ANXA2 expression on glioma aggressiveness, we firstly performed ANXA2 IHC on a series of 89 gliomas. IHC stainings disclosed that ANXA2 protein levels are significantly higher in GBM (*p* < 0.0001) compared to less aggressive tumors (Figure 1A–1B and Supplementary Figure S1). To validate our results, we next retrieved ANXA2 gene expression values from GSE4290 [24] and GSE7696 [25] glioma patients cohorts confirming a significant over-expression of ANXA2 transcript in gliomas relative to control tissues and its progressive increase with tumor grade (Figure 1C, 1D and Supplementary Table S1).

We then correlated ANXA2 IHC scores with clinical outcome of patients in terms of progression-free and overall survival (PFS and OS). In particular, glioma patients with “Very Low” ANXA2 IHC score (< 25° percentile) show a significantly prolonged PFS and OS when compared with remaining “ANXA2 High” patients (Table 1 and Supplementary Figure S2A–S2D). Since this result could be partially biased by an unbalanced distribution of low grade tumors (grade II-III and secondary) in the ANXA2 Very Low subgroup, we then analyzed the impact of ANXA2 IHC score only in GBM patients. Importantly, GBM patients with an ANXA2 Very Low score (< 25° percentile) display a significant increase in PFS and OS compared to all other GBMs (Figure 1E, 1F, Table 1 and Supplementary Figure S2E, S2F), thus strengthening the correlation of ANXA2 with GBM aggressiveness. In order to validate these results, we analyzed ANXA2 gene expression data from two independent cohorts of GBM patients (the TCGA dataset [26, 27] and GSE13041 [28]) and correlated its expression to patient outcome. Log-rank analysis confirmed that GBM patients expressing “Very Low” levels of ANXA2 mRNA (< 25° percentile) survived significantly longer in terms of OS (Figure 1G, 1H and Table 1) and PFS (Table 1 and Supplementary Figure S3), independently from the molecular subtype to which they were assigned according to the Verhaak classification [29] (Supplementary Figure S4).

**Table 1:** Summary of Log-rank analysis results on patients groups

Tumor type (origin of data)	Survival	ANXA2 status	Median Survival (months)	Log-rank (Mantel-Cox) *p* value	Hazard Ratio (logrank) High/Very Low
*Gliomas from IHC*	PFS	ANXA2 High ANXA2 Very Low	9.6 34.6	< 0.0001	3.888
	OS	ANXA2 High ANXA2 Very Low	22.9 N.D.	0.0282	3.018
*GBM from IHC*	PFS	ANXA2 High ANXA2 Very Low	8.4 29	0.0005	3.406
	OS	ANXA2 High ANXA2 Very Low	14.1 55.3	0.0354	2.671
*GBM from TCGA*	PFS	ANXA2 High ANXA2 Very Low	8.3 10.89	< 0.0001	1.651
	OS	ANXA2 High ANXA2 Very Low	12.6 17.76	< 0.0001	1.589
*GBM from GSE13041*	OS	ANXA2 High ANXA2 Very Low	12.5 16.6	0.0402	1.43

Then, we assessed the prognostic potential of ANXA2 IHC score in our cohort of patients (Supplementary Table S2) by Cox-regression (multivariate) analysis which demonstrated that ANXA2 IHC score is an independent prognostic factor for PFS (*p* = 0.041; Table 2). Intriguingly, when considering only GBMs, ANXA2 score retains an even stronger prognostic value for PFS (*p* = 0.029; Table 2).

**Table 2:** Multivariate analysis

	Progression free survival		Overall survival	
	Univariate (*p* value)	Multivariate (*p* value)	Univariate (*p* value)	Multivariate (*p* value)
*Variables*	Glioma (grade II-IV and secondary)			
*Sex*	0.268	0.606	0.016*	0.090
*Age (≤ 60; > 60 years)*	0.881	0.720	0.194	0.355
*Performance Score (≤ 1; > 1)*	0.004*	0.020*	< 0.001*	0.005*
*MGMT promoter (methylated or not)*	0.391	0.891	0.148	0.694
*IDH mutation*	0.026*	0.876	0.001*	0.968
*ANXA2 IHC score (≤ 4; > 4)*	0.001*	0.041*	0.016*	0.404
	**Glioblastoma (only grade IV)**			
*Sex*	0.836	0.555	0.107	0.106
*Age (≤ 60; > 60 years)*	0.878	0.498	0.867	0.564
*Performance Score (≤ 1; > 1)*	0.007*	0.054	< 0.001*	0.019*
*MGMT promoter (methylated or not)*	0.488	0.892	0.246	0.934
*IDH mutation*	0.230	0.679	0.049*	0.975
*ANXA2 IHC score (≤ 4; > 4)*	0.008*	0.029*	0.035*	0.607

* highlight significant *p* values < 0.05

### ANXA2 inhibition dramatically affects gene expression profile of GBM cells

**Figure 2:**
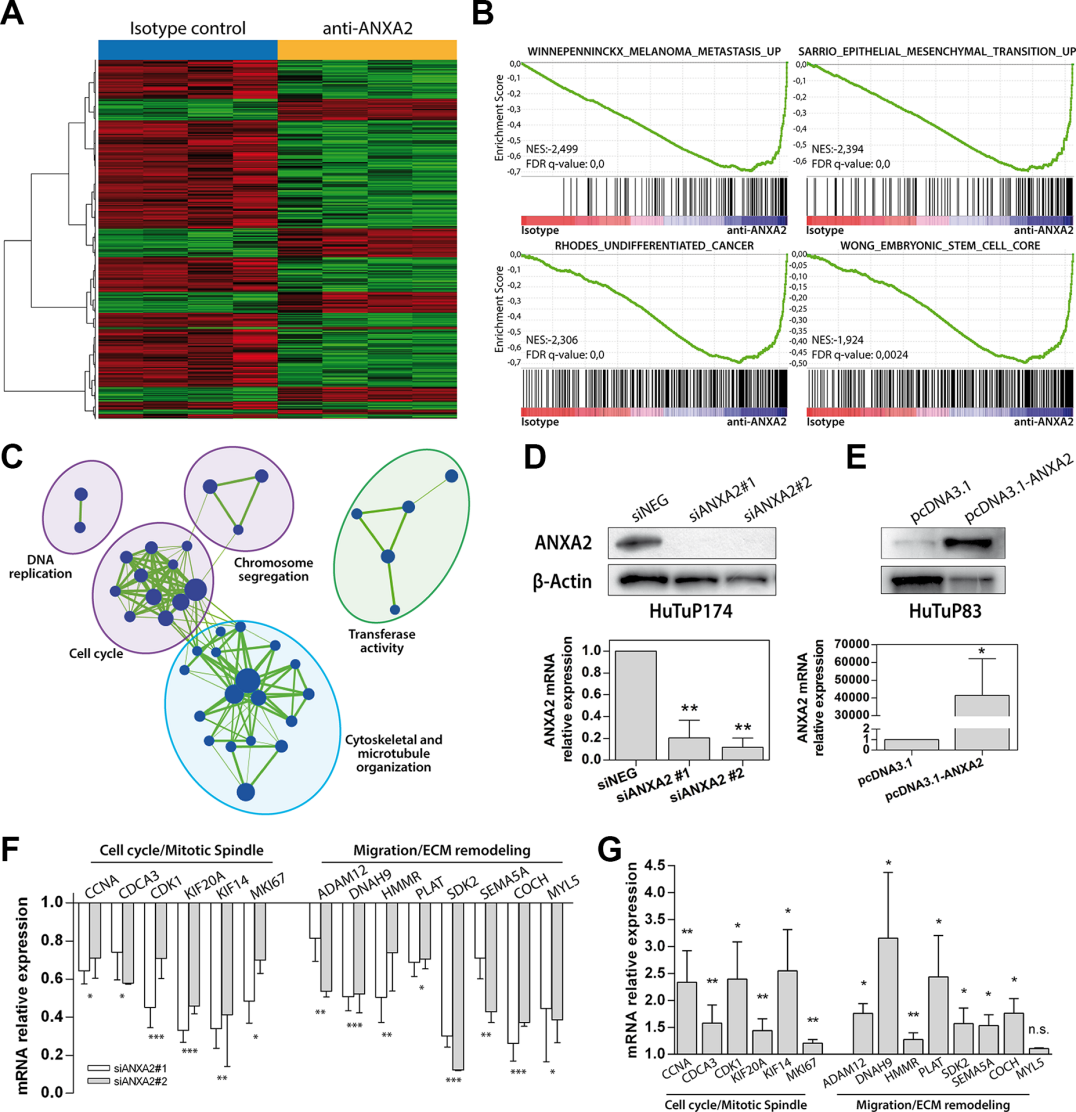
ANXA2 inhibition down-regulates the expression of genes involved in metastasis, EMT, cytoskeletal remodeling and cell cycle (**A**) Heat map generated by supervised analysis of HuTuP174 primary GBM cells treated with a monoclonal anti-ANXA2 antibody for 48 h (4 isotype vs. 4 ANXA2 antibody-treated cells) using the 855 differentially expressed probe sets (lFDR < 0.05). (**B**) Representative GSEA enrichment plots demonstrating that probes down-regulated after ANXA2 inhibition are enriched for genes involved in the metastatic and EMT processes (upper panels), and a stem-like/undifferentiated phenotype (lower panels). Plot were generated by c2 curated gene sets in the GSEA MSigDatabase. The green curves show the enrichment score and reflects the degree to which each gene (black vertical lines) is represented at the top or bottom of the ranked gene list. The heat map indicates the relative abundance (red to blue) of the genes specifically enriched in the anti-ANXA2-treated as compared with the isotype control-treated cells. (**C**) Enrichment Map based on results of c5 Gene Ontology GSEA-MSigDB, generated using Enrichment Map Cytoscape plug-in. Node represents the functional gene sets and the size is proportional to the size of gene set. Edge thickness is proportional to the overlap between the gene sets. We show only gene sets that are enriched with a fdr < 5% and only gene sets with a size between 15 and 500 genes were analyzed. Gene sets with common biological function are grouped in cluster and labeled with Gene Ontology terms. Green indicate negative enrichment in ANXA2 antibody-treated cells. (**D** and **E**) Evaluation of ANXA2 protein (upper panels) and mRNA (lower panels) expression in GBM cells after ANXA2 gene silencing (two different siRNAs against ANXA2 mRNA vs. siNEG in HuTuP174 GBM cells) or ANXA2 over-expression (HuTuP83) respectively by transient transfection procedures. (**F** and **G**) Validation of the transcriptional expression of a series of genes, selected from down-regulated genes after anti-ANXA2 treated cells, involved in cell cycle (*CCNA*, *CDCA3*, *CDK1*, *KIF20A*, *KIF14* and *MKI67*) and cell migration (*ADAM12*, *DNAH9*, *HMMR*, *PLAT*, *SDK2*, *SEMA5A*, *COCH*, *MYL5*) cellular processes in ANXA2 silenced (HuTuP174) and ANXA2 over-expressing (HuTuP83) GBM cells respectively. *p < 0.05, **p < 0.01, ***p < 0.001, n.s. not significant by one-way ANOVA or paired *t*-test.

Starting from previous results, we analyzed TCGA and GSE13041 datasets in order to compare the gene expression profile of ANXA2 Very Low and ANXA2 High GBMs. We identified 421 up-regulated and 298 down-regulated genes in common between the two cohorts of patients and significantly associated to an “ANXA2-high expression phenotype” (differentially expressed genes between ANXA2 High versus ANXA2 Low tumors with 25° percentile of ANXA2 expression as cut-off; Supplementary Figure S5A and Supplementary Table S3). Interestingly, Gene Set Enrichment Analysis (GSEA) of differentially expressed genes revealed a positive enrichment for cell migration and epithelial to mesenchymal transition (EMT) signatures in ANXA2 High GBMs (Supplementary Figure S5B). Moreover, it showed ANXA2 High GBMs as positively and negatively enriched for genes related to the “Mesenchymal” and “Proneural” molecular subtypes respectively (Supplementary Figure S5B). In order to better characterize the link between ANXA2 levels and GBM transcriptional profile, we retrieved gene expression data from GBM cells treated with an ANXA2 neutralizing antibody, previously reported to efficiently inhibit ANXA2 activity [30, 31]. To this end, we derived a series of primary GBM cultures from patient biopsies (Supplementary Table S4) and selected ANXA2 highly expressing GBM cells by WB (ANXA2^hi^; Supplementary Figure S6). ANXA2^hi^ cells were then treated with the ANXA2-neutralizing antibody and their transcriptional profile analyzed by Affymetrix chips. Supervised analysis retrieved 855 differentially expressed probes between anti-ANXA2 and Isotype control-treated GBM cells (634 down- and 221 up-regulated; Figure 2A and Supplementary Table S5). Interestingly, ANXA2-inhibited cells showed a negative enrichment for EMT and metastasis genes (Figure 2B), confirming previous results from TCGA and GSE13041 datasets (Supplementary Figure S5). Moreover, anti-ANXA2-treated cells were negatively enriched for genes correlated to a “stem cell/undifferentiated phenotype”, suggesting that ANXA2 modulation would potentially impact also cellular differentiation (Figure 2B lower panels).

To account for further effects mediated by ANXA2, we generated an enrichment map based on gene ontology (GO) analysis of differentially expressed genes, which clearly showed that ANXA2 blockade is sufficient to significantly down-regulate genes clustering in cell cycle, DNA replication, chromosome segregation and microtubule organization gene families, thus pointing ANXA2 also as a potential modulator of GBM cell proliferation (Figure 2C). Analysis of up-regulated genes revealed no association to specific enrichment modules or gene sets; however, GO analysis showed a general up-regulation of genes actively involved in the control of the transcriptional machinery (data not shown). Finally, to account for a potential molecular subtype shift after ANXA2 inhibition, HuTuP174 primary GBM cells (isotype- or ANXA2 antibody-treated) were assigned to their specific molecular subtype according to the Verhaak classifier [29]. As a result, HuTuP174 were classified as “classical”, without shifting their assigned subtype after ANXA2 inhibition (data not shown).

To validate these data we evaluated the expression of selected genes in GBM cells silenced for ANXA2 (Figure 2D) or transiently over-expressing ANXA2 mRNA (Figure 2E). As a result, ANXA2 silencing was able to dramatically down-regulate a series of genes down-modulated by ANXA2 antibody treatment (Supplementary Table S5) and particularly involved in the regulation of cell cycle, cell migration and ECM remodeling (Figure 2F). Conversely, the expression of these genes was significantly augmented by ANXA2 over-expression in ANXA2^low^ cells (Figure 2G).

### GBM cell migration and invasion are sustained by ANXA2

In order to functionally validate the role of ANXA2 as master sustainer of GBM cell dissemination, we modulated ANXA2 activity/expression in primary cells (Supplementary Table S4) and evaluated their migratory and invasive properties.

**Figure 3:**
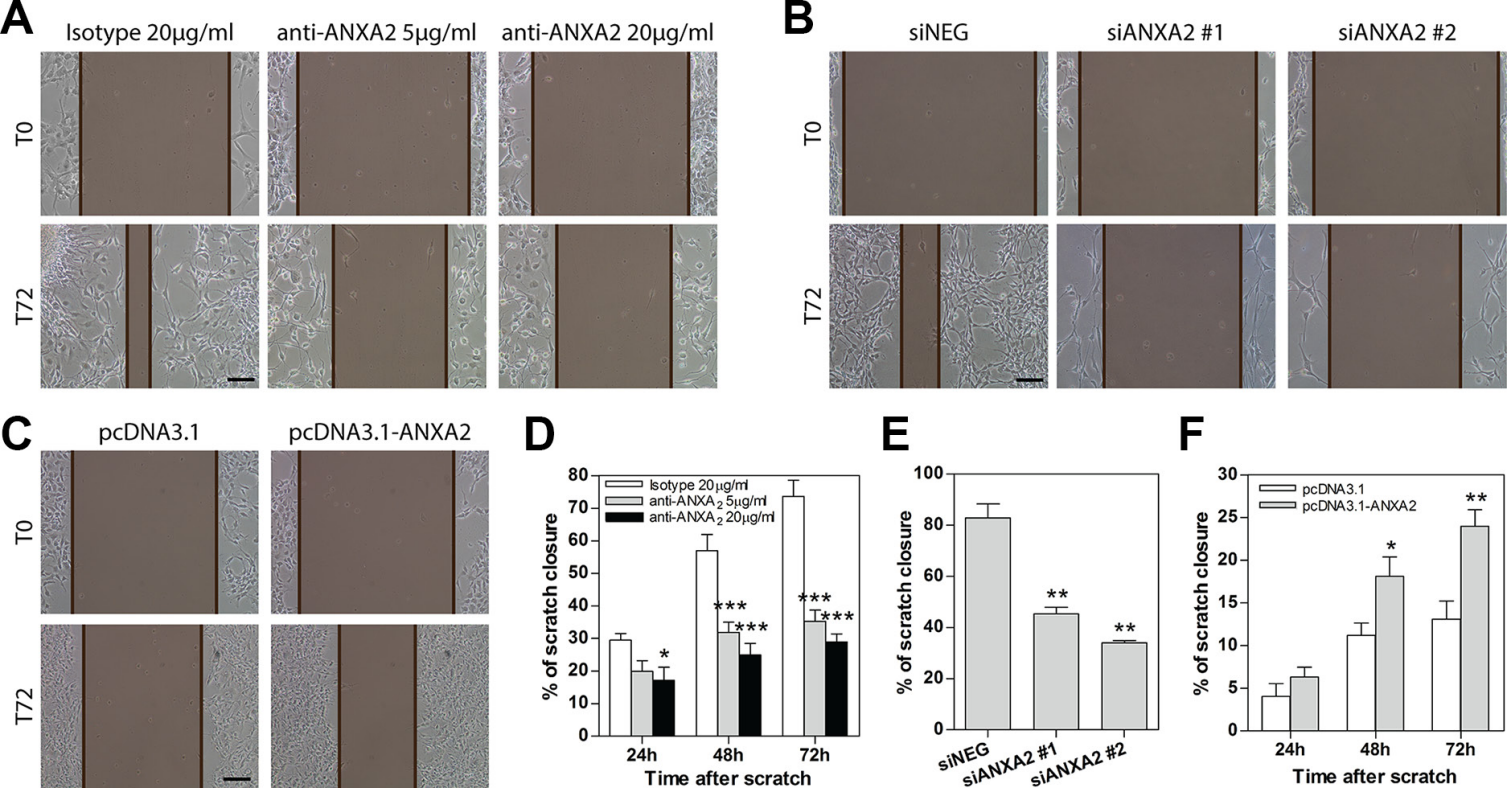
Modulation of ANXA2 activity or expression levels impacts primary GBM cell migration (**A**–**C**) Representative images showing the ability of GBM cells to close the wound within 72 h after scratching the cell monolayer during scratch assays. These have been performed on primary GBM cells treated with a monoclonal anti-ANXA2 antibody (A; HuTuP175), silenced for ANXA2 (B; HuTuP174) or ANXA2 over-expressing (C; HuTuP83) cells. The distance between the two edges of the scratch is marked in brown and has been quantified by using Adobe Photoshop CS6. Original magnification 10x; bar:50 μm. (**D–F**) Bar graphs showing relative quantification of the distance between scratch edges in anti-ANXA2 antibody-treated (D; *N* = 6 for HuTuP107, HuTuP174 and HuTuP175), ANXA2-silenced (E; *N* = 3 for HuTuP174) or ANXA2 over-expressing (F; *N* = 3 for HuTuP83) primary GBM cells at the indicated timepoints. The migration ability of GBM cells after ANXA2 silencing is reported only at 72 h. *p < 0.05; **p < 0.01; ***p < 0.001 by one-way ANOVA or paired *t*-test.

Inhibition of ANXA2 by neutralizing antibody resulted in a dramatic impairment of GBM cell migration during scratch assays. In particular, the inhibitory effect on cell migration was detectable early after treatment (24 h), being progressively stronger at later timepoints (Figure 3A, 3D and Supplementary Figure S7A). This effect was confirmed also by ANXA2 silencing (Figure 3B, 3E). In both conditions, we did not observe impairment of GBM cell viability (data not shown). Conversely, ANXA2 over-expression significantly enhanced the migratory properties of cells endowed with low motility (Figure 3C, 3F and Supplementary Figure S7B). Inhibition of ANXA2 by antibody prevented scratch closure also of normal sub-ventricular zone (SVZ)-derived stem cells (SC23; Supplementary Figure 7C, 7D), nevertheless without reducing their viability (data not shown).

**Figure 4:**
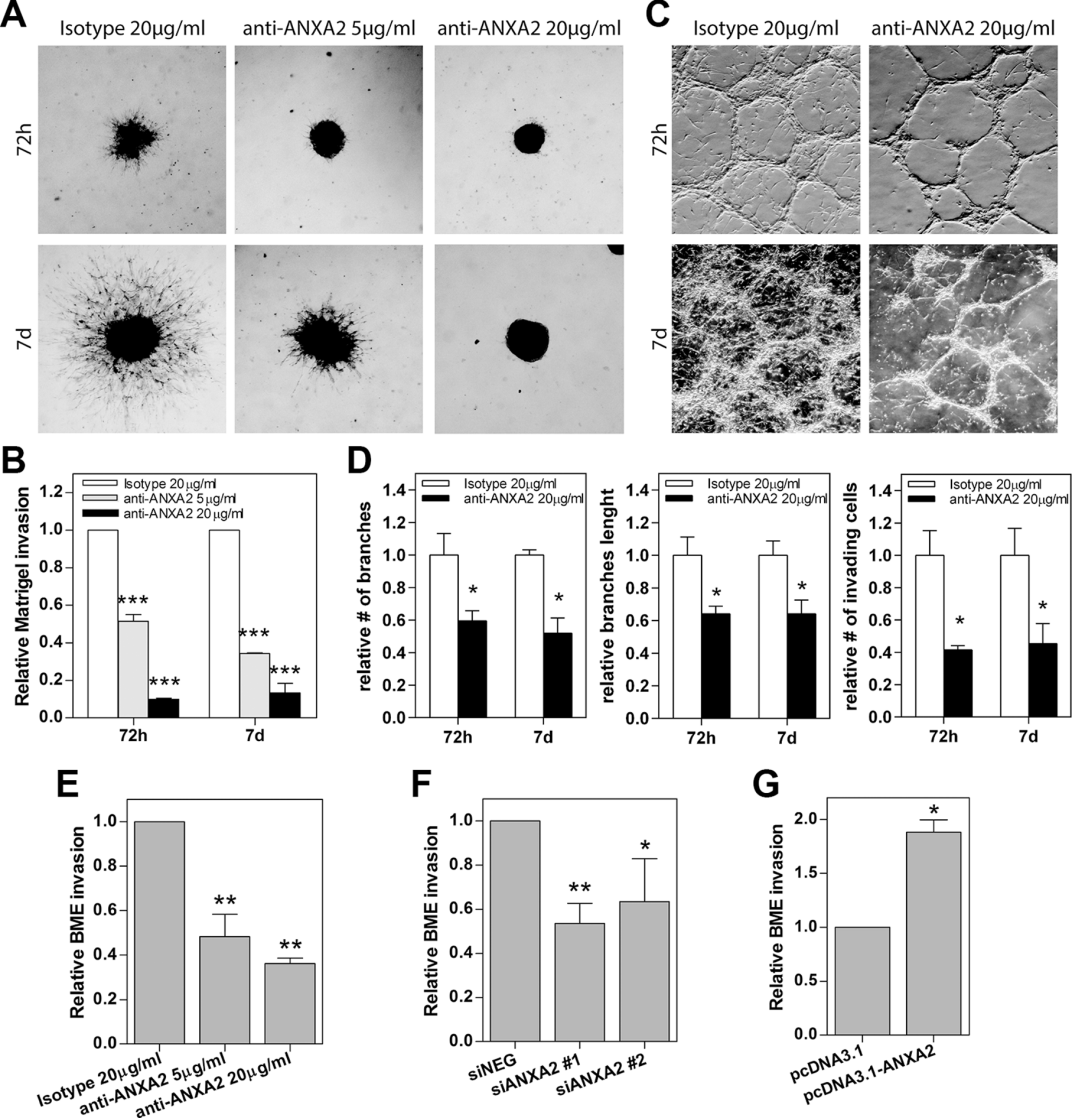
Modulation of ANXA2 activity or expression levels impacts primary GBM cell invasion (**A** and **C**) Representative images of primary GBM cells growing as spheres (HuTuP174) or as reticulates (HuTuP13) on Matrigel-coated dishes and treated with anti-ANXA2 antibody until the indicated timepoints. Original magnification 4x. (**B**) Relative quantification of the length of the protrusions invading the matrix and spreading away from the spheres showed in (A). (**D**) Relative quantification of the length and number of cell reticulate branches and the number of invading cells showed in (B). Bar graphs showing relative invasion of primary GBM cells after ANXA2 inhibition (**E**) *N* = 3 for HuTuP13 and HuTuP175), gene silencing (**F**) *N* = 3 for HuTuP13 and HuTuP174) or over-expression (^**G**^) *N* = 4 for HuTuP83) as quantified by Cultrecoats^®^ BME-based assays as described in the Methods section. *p < 0.05; **p < 0.01; ***p < 0.001 by one-way ANOVA or paired *t*-test.

We then tested the invasion ability of ANXA2^hi^ cells in a basement membrane-like matrigel assay with cells plated on the top of a thin layer of semisolid medium. In this condition, HuTuP174 GBM cells grew as clonal spheres and inhibition of ANXA2 by antibody treatment counteracted their invasive properties in a dose dependent manner (Figure 4A, 4B). Importantly, the highest dose of antibody completely halted cell invasion until one week after treatment, with GBM cells being restricted in the spheres without any spreading (Figure 4A, 4B). We then confirmed these data in two additional primary GBM cultures endowed with reticulate growth (HuTuP13 and 176) which showed a dramatic reduction of the number and length of branches and the amount of invading cells (Figure 4C, 4D and Supplementary Figure S8A). As a further validation, ANXA2 inhibition/silencing were both able to halve the number of ANXA2^hi^ GBM cells able to pass through a basal membrane extract (BME)-coated transwell (CultreCoat^®^Cell Invasion assay) within 48 h (Figure 4E, 4F and Supplementary Figure S8B, S8C). Moreover, ANXA2 over-expression significantly increased GBM cell invasion (Figure 4G and Supplementary Figure S8D).

**Figure 5:**
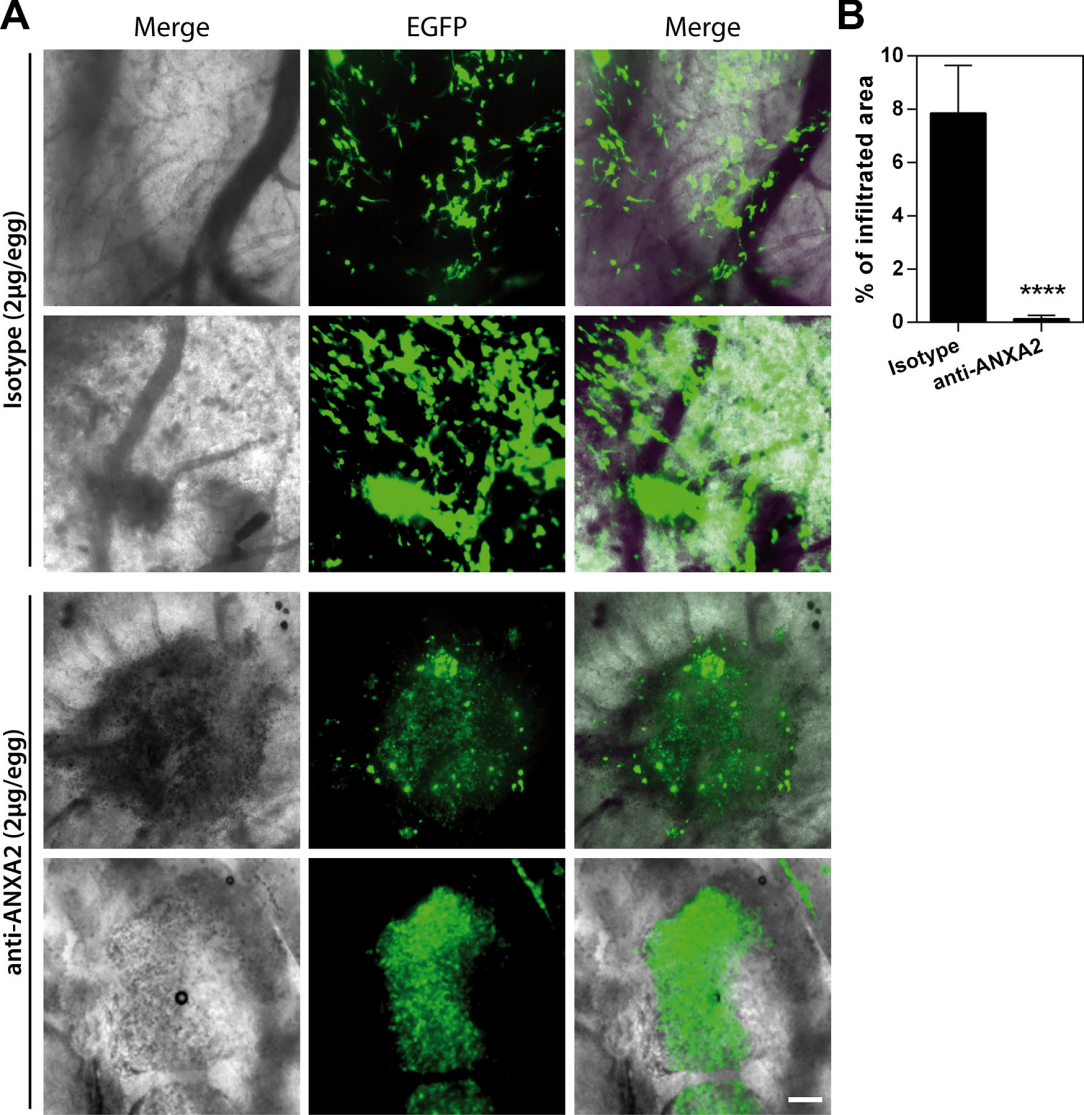
ANXA2 inhibition reduces GBM cell dissemination *in vivo* in the CAM invasion assay (**A** and **B**) Representative images showing the migration/invasion of HuTuP13-EGFP primary GBM cells (green) inside the observation area after 3 days of treatment with 2 μg of isotype control (upper panels) or ANXA2 antibody (lower panels) (2 μg/egg/day) (A) and quantification of the area covered by cells relative to the entire observation space (24,7 mm^2^; B). Original magnification 20x; bar:200 μm. ****p < 0.0001 by *t*-test.

To definitely assess the inhibitory effects exerted by ANXA2 blockade on the dissemination of GBM cells, we analyzed the impact of ANXA2 antibody treatment *in vivo* on GBM primary cells (EGFP expressing HuTuP13 cells) xeno-transplanted in the chick embryo chorioallantoic membrane (CAM). After 72 h from initial treatment (2 μg antibody/egg/day), isotype treated cells actively dispersed in the CAM, moving away from the initial site of cell deposition (Figure 5A, upper panels). On the contrary, ANXA2 inhibited cells formed restricted cellular masses, confined in the deposition site, without any spreading (Figure 5A, lower panels). As a result, control cells disseminated covering a 60-fold larger area than ANXA2 antibody treated cells (Figure 5B), demonstrating that ANXA2 inhibition is able to completely block GBM cell invasiveness also in an *in vivo* setting.

**Figure 6:**
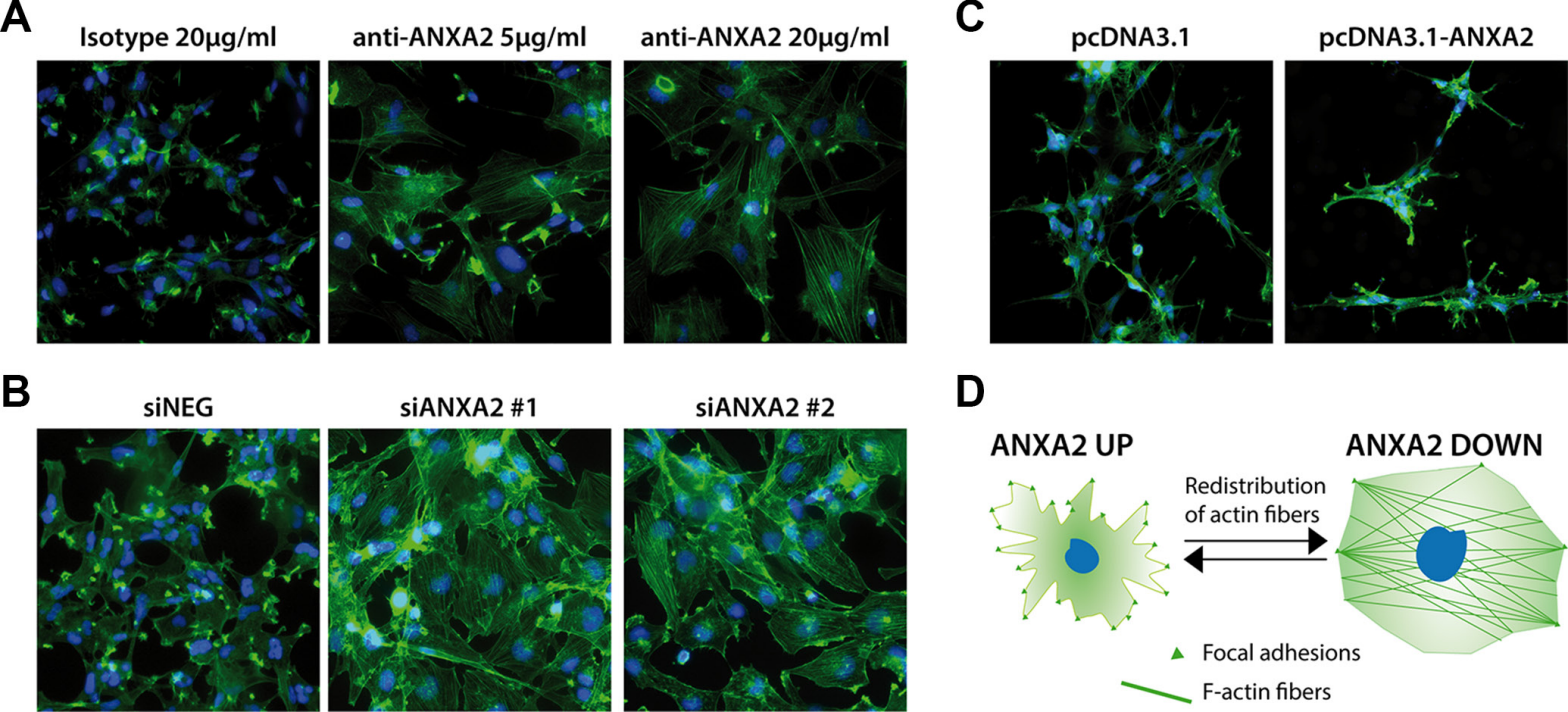
Modulation of ANXA2 levels is accompanied by dramatic cytoskeletal remodeling (**A–C**) Representative immunofluorescence images of GBM cells after ANXA2 inhibition, gene silencing or over-expression respectively and stained with a FITC-labeled phalloidin probe in order to reveal the distribution of F-actin (green). Cell nuclei have been counterstained with DAPI (blue). Original magnification 20x. (**D**) Cartoon resembling the major morphological and cytoskeletal changes associated to the modulation of ANXA2 in GBM cells.

Cellular migration/invasion is usually mediated by re-organization of cytoskeletal actin fibers and their link to ECM through focal adhesions (FAs) [32, 33]. In this context, it has been previously reported that ANXA2 binds to filamentous (F)-actin [31]. For this reason, we analyzed the possible involvement of ANXA2 in cytoskeletal remodeling, as suggested by GO analysis (Figure 2C). Phalloidin staining revealed that ANXA2^hi^ GBM cells are characterized by cytoskeletal fibers assembled in FA-like structures, indicative of a migrating phenotype (Figure 6A, 6B, 6D). Conversely, ANXA2 inhibited/silenced cells displayed a dramatic redistribution of F-actin fibers and an almost complete loss of FAs (Figure 6A, 6B, 6D), without affecting distribution of microtubules (data not shown). ANXA2 over-expression in ANXA2^low^ GBM cells increased the number of FAs (Figure 6C, 6D), thus reinforcing the hypothesis of ANXA2 as a major player in the control of cytoskeletal dynamics.

### ANXA2 impairment induces differentiation and inhibits proliferation of GBM cells

**Figure 7:**
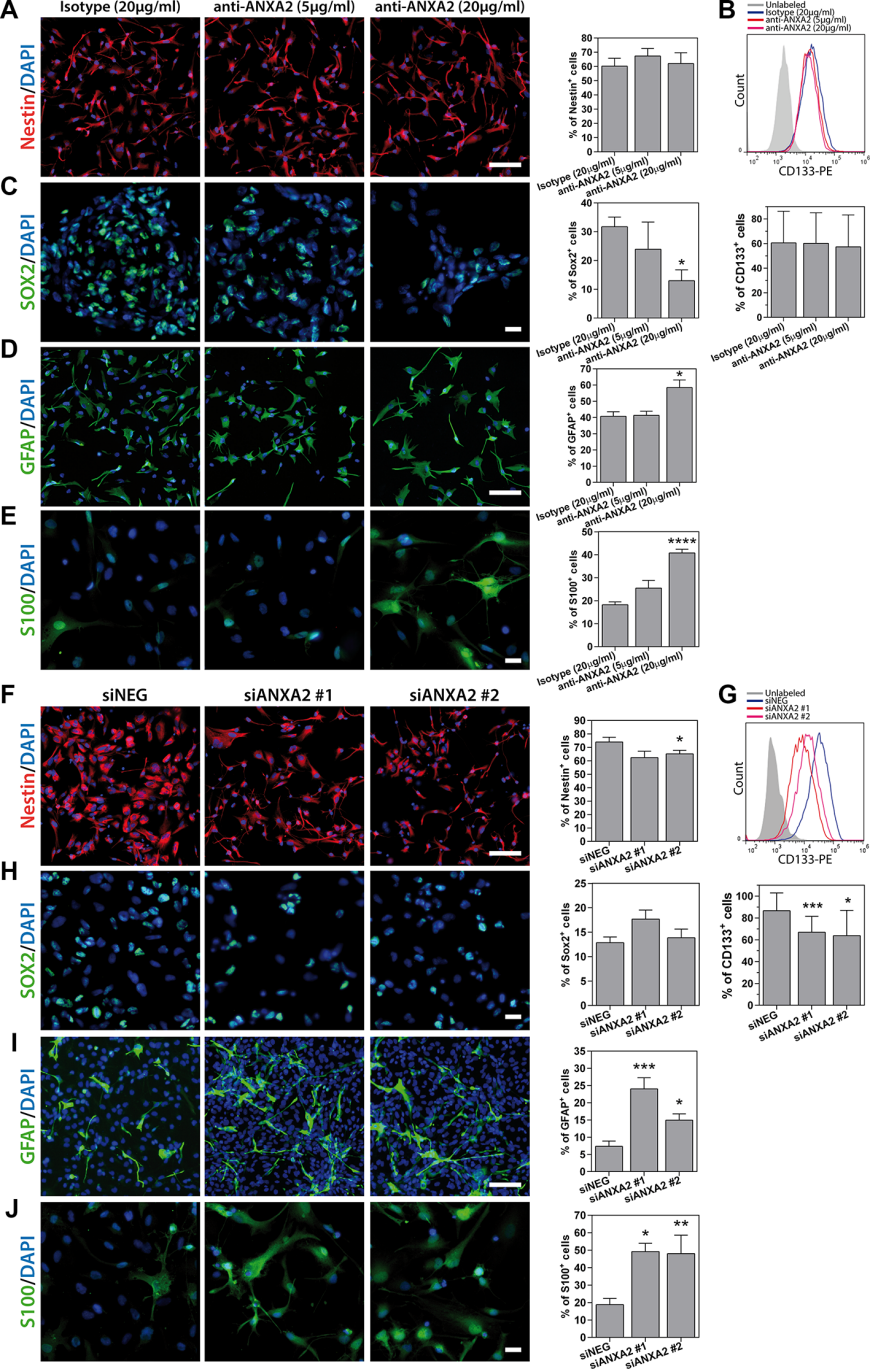
Analysis of primary GBM cell phenotype after ANXA2 inhibition or gene silencing (**A–E**) Representative immunofluorescence images showing primary GBM cells (HuTuP175) treated with anti-ANXA2 monoclonal antibody or isotype control antibody and stained with anti-Nestin (A; red), anti-Sox2 (C; green), anti-GFAP (D; green) or anti-S100 (E; green) antibodies, and relative quantification of positive cells (right panels). (B) Representative plot showing overlay of CD133^+^ cell populations in control and ANXA2 antibody treated cells (HuTuP13; upper panel) and relative quantification (*N* = 5 for HuTuP13, HuTuP82 and HuTuP175; lower panel). (**F–J**) Representative immunofluorescence images showing primary GBM cells (HuTuP13) silenced for ANXA2 and stained with anti-Nestin (F; red) or anti-Sox2 (H; green), anti-GFAP (I; green) or anti-S100 (J; green) antibodies and relative quantification of positive cells (right panels). (G) Representative plot showing overlay of CD133^+^ cell populations in siNEG and siANXA2 transfected cells (HuTuP13; upper panel) and relative quantification (*N* = 9 for HuTuP13 and HuTuP82; lower panel). For all stainings GBM cell nuclei have been counterstained with DAPI (blue). Percentages of positive cells have been calculated as number of positive cells/number of DAPI^+^ nuclei per microscopic field. At least 8 fields per condition have been analyzed. Original magnification 10–20x. Bar:50 μm. *p < 0.05;***p < 0.001 by one-way ANOVA analysis. Significance is reported relative to control (isotype treated or siNEG transfected) GBM cells.

**Figure 8:**
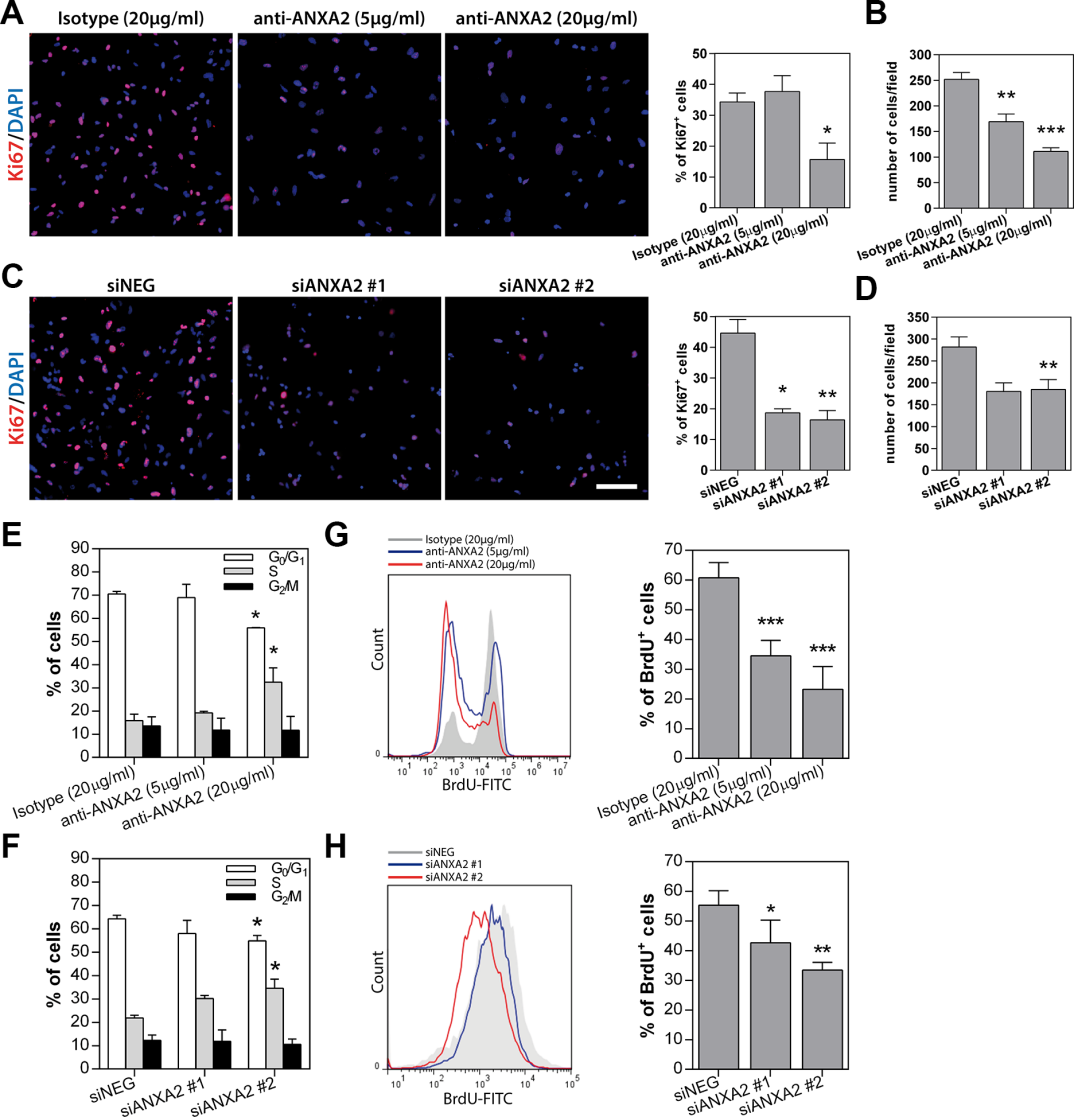
ANXA2 inhibition or gene silencing strongly reduces GBM cell proliferation by inducing a partial block at the S-G_2_/M checkpoint (**A–D**) Representative immunofluorescence images of primary GBM cells treated with anti-ANXA2 antibody (HuTuP175) or silenced for ANXA2 (HuTuP13) and stained with anti-Ki67 (red) monoclonal antibody. Right panels show relative quantification of Ki67^+^ cells (A and C) or quantification of DAPI^+^ nuclei per microscopic field depending on the experimental condition (B and D). Percentages of Ki67^+^ cells have been calculated as number of positive cells/number of DAPI^+^ nuclei per microscopic field. At least 8 fields per condition have been analyzed. Original magnification 10x. Bar:50 μm (**E** and **F**) Bar graph summarizing cell cycle analyses of ANXA2-neutralized or –silenced GBM cells (HuTuP13, HuTuP82 and HuTuP174). (**G** and **H**) Representative graphs showing BrdU incorporation analysis performed on HuTuP174 GBM cells after anti-ANXA2 antibody treatment or HuTuP53 GBM cells after ANXA2 silencing respectively. Right panels show bar graphs reporting relative quantifications. BrdU analyses have been performed on HuTuP13, HuTuP53, HuTuP82 and HuTuP174 primary GBM cells. *p < 0.05; **p < 0.01; ***p < 0.001 by one-way ANOVA. Significance is reported relative to control (isotype treated or siNEG transfected) GBM cells.

GSEA and GO analysis suggested a potential role of ANXA2 in the control of cell phenotype and proliferation (Figure 2B, 2C). According to this hypothesis, ANXA2 blockade significantly reduced the number of cells expressing the stem cell marker Sox2, nevertheless without affecting the expression of Nestin and CD133 (Figure 7A–7C). Conversely, ANXA2 inhibition was responsible of a significant increase of the number of cells expressing the astrocytic differentiation markers GFAP and S100 (Figure 7D, 7E and Supplementary Figure S9A). Nevertheless, we did not observe induction of cell differentiation toward other neural lineages such as neurons or oligodendrocytes, since examined cells showed very low or absent expression of the neuronal marker microtubule associated protein 2 (MAP2; Supplementary Figure S9B) and oligodendrocyte specific protein (OSP; Supplementary Figure S9C). These effects increased in ANXA2-silenced GBM cells, which displayed a significant reduction of Nestin^+^ and CD133^+^ cells instead of Sox2 (Figure 7F–7J and Supplementary Figure S9D, S9E). In line with these results, ANXA2 blockade/silencing strongly inhibited GBM cell proliferation. Indeed, the number of cells and the expression of the proliferation marker Ki67 were significantly reduced after ANXA2 knockdown (Figure 8A–8D and Supplementary Figure S10). To better characterize these effects, we performed a PI-based cell cycle analysis on both antibody treated and silenced cells. We measured a significant reduction of cells in the G1 phase and a parallel accumulation of cells in the S phase of the cell cycle (Figure 8E, 8F), suggesting that impairment of ANXA2 is sufficient to partially arrest GBM cells at the S-G2/M cell cycle checkpoint. As a confirmation, experiments of BrdU uptake clearly showed a significant decrease of its incorporation within 96 h in the same cells (Figure 8G, 8H). On the other hand, ANXA2 over-expression did not affect neither phenotype, nor cell cycle dynamics of GBM cells (data not shown), suggesting that ANXA2 would be necessary for the maintenance of a proliferative and in some way “less differentiated” cell phenotype, but its accumulation is not sufficient to perturb these systems by itself.

### An ANXA2^down^ signature predicts GBM patient survival

**Figure 9:**
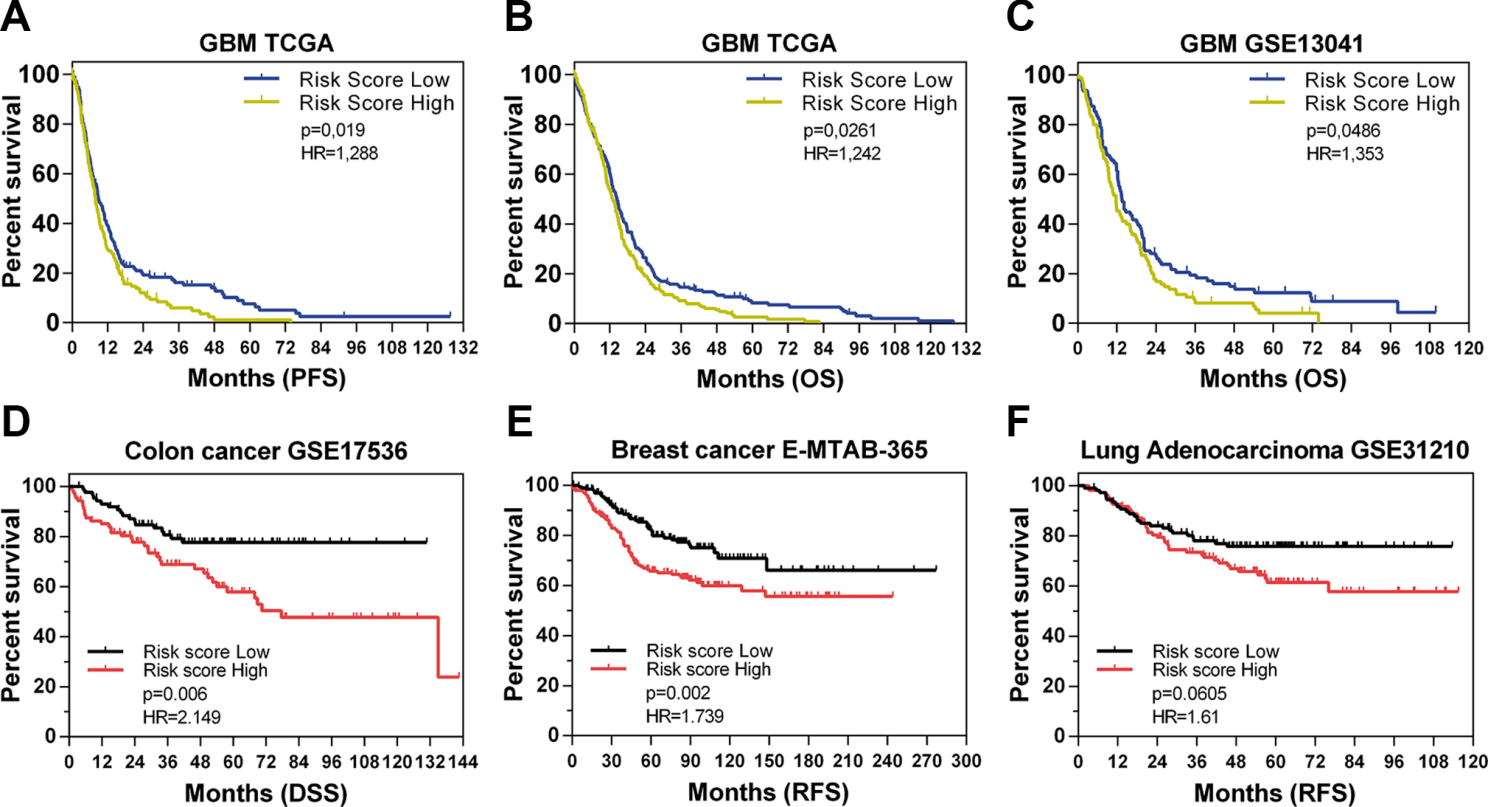
The ANXA2^down^ signature predicts GBM patient survival (**A–C**) Kaplan Meier curves showing the prognostic potential of the ANXA2^down^ gene signature applied on GBM patients from TCGA (PFS and OS in A and B respectively) and the GSE13041 (OS in C) datasets. (**D–F**) Kaplan Meier curves showing the application of the ANXA2^down^ gene signature on different solid tumors datasets including GSE17536 (disease specific survival-DSS in colon cancer patients in D), E-MTAB-365 (relapse free survival-RFS of breast cancer patients in E) and GSE31210 (RFS of lung adenocarcinoma patients in F). Hazard Ratios have been calculated as Risk Score Low/High.

To finally test the clinical relevance of the multiple effects exerted by ANXA2 modulation, we investigated the correlation of an ANXA2-dependent gene signature (ANXA2^down^), with clinical outcome in TCGA and GSE13041 datasets. We used the ANXA2^down^ gene signature, based on the most down-regulated probes after ANXA2 antibody treatment, to divide patients into two equal groups on the basis of the median expression of the signature in the bulk GBM tumors. We observed a significant positive correlation between ANXA2^down^ signature and survival (PFS and OS) in GBM patients from both datasets (Figure 9A–9C and Supplementary Table S6). In addition, we analyzed the potential correlation of the ANXA^down^ signature with clinical outcome also in other publicly available solid tumor datasets including colon cancer (GSE17536; [34]), breast cancer (E-MTAB-365; [35]) and lung adenocarcinoma (GSE31210; [36]). Of note, the signature significantly correlated with disease-specific (DSS) and relapse-free survival (RFS) in colon and breast cancer respectively (Figure 9D, 9E), showing a partial association with RFS also in lung adenocarcinoma (Figure 9F). These findings indicate that genes directly or indirectly regulated by ANXA2 retain a consistent prognostic relevance and that ANXA2 is endowed with the ability to participate in multiple cancer processes, fundamental for tumor survival, thus being a potential therapeutic target in GBM.

## DISCUSSION

Most GBM tumors recur within 2 cm from the original tumor margin. This peculiar characteristic of GBM relay on brain cancer cell ability to invade the surrounding normal brain tissues, thus escaping from surgical removal and localized radiotherapy [37]. In this context, we and others recently exploited 5-ALA in order to properly identify and characterize dispersed GBM cells at the tumor margin, demonstrating that some of these cells are still endowed with stem-like characteristics, potentially hampering treatments [38–40]. In this discouraging landscape, a more comprehensive knowledge of the mechanisms involved in GBM cell spreading becomes particularly relevant.

Agreeing to this view, we chose to elucidate the potential involvement of Annexin A2 in regulating GBM cell dissemination. Indeed, ANXA2 has been proved to sustain EMT and invasion of pancreatic ductal adenocarcinoma [41], induce hepatocellular carcinoma and breast cancer metastasis [20, 42] and was found up-regulated in highly invasive carcinomas [43–45]. Moreover, ANXA2 has been correlated to the observed hyperfibrinolysis-dependent bleeding in acute promyelocytic leukemia [46].

Here, we show that ANXA2 is significantly over-expressed in GBM in three independent cohorts of patients (Figure 1A–1D) and that ANXA2 expression can be considered as an independent prognostic factor in glioma [31, 47–49]. More importantly, we demonstrate that low/absent expression of ANXA2 identifies a subgroup of GBM patients endowed with better prognosis in three different cohorts of GBM patients (Figure 1E–1H), with ANXA2 IHC score retaining a strong prognostic value for PFS in multivariate analysis (Table 2).

Since ANXA2 has been involved in multiple cellular functions including vesicle trafficking, cell division, calcium signalling and cellular growth [21], we analyzed a gene expression profile correlated to low ANXA2 expression in GBM tumors and characterized the transcriptional changes associated to its inhibition in GBM cells *in vitro*. Importantly, besides its reported role in cell migration and invasion, GSEA pointed out the existence of a positive and negative correlation between genes associated to high ANXA2 expression and the “Mesenchymal” and “Proneural” molecular subtypes [29] respectively. This information is particularly relevant, since GBMs belonging to the Proneural subtype display clinical and molecular features often associated to less aggressive tumors and long surviving patients [29, 50–53]. On the other hand, tumors from the Mesenchymal molecular subgroup are characterized by high expression of EMT markers such as MET and CD44, reminiscent of de-differentiated aggressive tumors [29]. We also confirmed these findings by GSEA of ANXA2-inhibited GBM cells, which shows a significant negative enrichment of gene signatures associated to undifferentiated cancer and stem cell phenotype (Figure 2B), suggesting the potential involvement of ANXA2 also as a modulator of differentiation in GBM cells. Interestingly, we also examined the effects produced by intensive treatment protocols (defined as more than one cycle of single or concurrent chemo- and radiotherapy) in GBM patient subgroups generated on the basis of ANXA2 expression in the TCGA dataset. Our analyses show that aggressive treatment prolonged survival only in ANXA2 High tumors (HR = 0,475; Supplementary Figure S11A) rather than ANXA2 Very Low GBMs (HR = 0,74; Supplementary Figure S11B), resembling previous data on the Mesenchymal or Proneural subclasses respectively [29]. These latter results underline the potential and visionary clinical relevance of the assessment of ANXA2 expression in suggesting differential therapeutic strategies in GBM.

All these analyses recreate a complex scenario in which ANXA2 seems to sustain GBM cell aggressiveness at multiple levels. In addition, enrichment map generated by GO analysis of differentially expressed genes upon ANXA2 inhibition, points out a potential participation of ANXA2 also in the regulation of cytoskeletal organization and cell cycle dynamics (Figure 2C). Here we show that reduction of ANXA2 activity is sufficient to: i) dramatically impair GBM cell invasiveness; ii) induce a strong rearrangement of cytoskeletal structures; iii) inhibit cell proliferation.

Although studies already reported that ANXA2 knockdown negatively affects invasiveness and proliferation of human and rodent glioma cell lines *in vitro* [54] and *in vivo* [31], to the best of our knowledge, we report for the first time the effects mediated by ANXA2 inhibition in patient-derived primary GBM cells, thus strengthening our conclusions. In particular, our data clearly indicate that ANXA2 down-modulation significantly reduces the expression of genes involved in DNA replication and chromosome segregation (Figure 2C, 2F, 2G), thus impairing GBM cell proliferation at the S-G_2_/M transition (Figure 8E–8H). This potential mechanism is added to different previous studies showing that ANXA2-mediated cell cycle effects could be attributed to p53 over-expression [55], Stat3 inhibition [56] or c-Myc-dependent cyclin D1 transcription [57]. Interestingly, ANXA2 over-expression did not alter GBM cell proliferation in our setting, suggesting that an intact ANXA2 function is necessary to sustain the complete oncogenic program engaged by GBM, but its up-regulation is not sufficient to trigger proliferation or activate a de-differentiation program by itself.

In a previous study, Rescher *et al.* demonstrated that insulin stimulation of normal BHK cells induced a massive rearrangement of actin fibers accompanied by cell spreading and detachment, and promoted a strong accumulation of F-actin and ANXA2 into FA-like structures at the cell periphery [58]. Authors proposed an ANXA2-mediated Rho/ROCK pathway control as the major responsible for the observed cytoskeletal remodeling. Our results are in line with these previous findings, showing that ANXA2^hi^ cells organize actin fibers into FA-like structures at the cell periphery; conversely, ANXA2 inhibited/silenced GBM cells acquire a less “contracting” and more flattened cell shape (Figure 6A–6D). Moreover, it has recently been reported that ANXA2 should control the invasive properties of glioma cells by a double mechanism consisting of: i) augmented cancer cell binding to endothelial cells that eases the process of vascular co-option; ii) increase of VEGF and PDGF production which induce angiogenesis [59]. These data add further evidence to the multiple invasion mechanisms reported for ANXA2.

Finally, based on gene expression data of ANXA2 neutralized cells, we were able to test the prognostic potential of an ANXA2^down^ signature in multiple cancer datasets, demonstrating that expression of genes regulated (most likely indirectly) by ANXA2 fluctuations predict cancer patients outcome by themselves (Figure 9A–9F). These results are particularly relevant since they allow to generalize the prognostic potential of ANXA2 and/or ANXA2-modulated transcripts to multiple solid tumors, thus highlighting its relevance as a master controller of cancer cell dissemination and metastasis. In this context, increasing evidence suggests the participation of ANXA2 in regulating the localisation as well as translation of specific transcripts. Indeed, specific ANXA2 domains are reported to bind to the 3′-UTRs of c-myc [60], collagen prolyl 4-hydroxylase-α(I) (C-P4H) [61] and N-methyl-D-aspartate R1 (NMDA-R1) [62] mRNAs, contributing to the post-transcriptional regulation of particular genes [63]. Therefore, a better comprehension of the direct or indirect effects mediated by ANXA2 on ECM degradation, cytoskeletal remodeling and gene transcription, will inevitably lead ANXA2 as future marker to be assessed for GBM management or to be targeted to inhibit dissemination.

## MATERIALS AND METHODS

### Neurosurgical sample collection, isolation and gas-controlled expansion of GBM cells

Written informed consent for the donation of adult tumor brain tissues was obtained from patients before tissue collection under the auspices of the protocol for the acquisition of human brain tissues obtained from the Ethical Committee of the Padova University-Hospital. All tissues were acquired following the tenets of the Declaration of Helsinki.

For this study we used GBM specimens isolated from 89 tumors taken at surgery (Supplementary Table S2) and then formalin-fixed and paraffin embedded (FFPE) for subsequent IHC analysis. Moreover, 8 primary GBM cultures have been used for *in vitro* experiments. General characteristics of patients from which we derived GBM primary cells are listed in Supplementary Table S4. Primary GBM cells were isolated and maintained in culture as described previously [64]. Briefly, tumor biopsies were subjected to mechanical dissociation and the resulting cell suspension was cultured on fibronectin-coated dishes in DMEM/F12 medium supplemented with BIT9500 (Stemcell Technologies Inc., Vancouver, Canada), 20ng/ml basic Fibroblast Growth Factor (bFGF; Sigma-Aldrich S.r.l., Milan, Italy) and 20ng/ml Epidermal Growth Factor (EGF; R&D Systems, Minneapolis, MN). GBM cells were maintained in an atmosphere of 2% oxygen, 5% carbon dioxide and balanced nitrogen in a Ruskinn C300 system for a proper cell expansion in hypoxic conditions (Ruskinn Technology Ltd, Bridgend, UK). SVZ-derived SC23 normal cells [65] were cultured in the same conditions as GBM primary cells. Cells were not cultured for more than 8 passages *in vitro* in order to avoid long term culture related effects. In some experiments, GBM cells were treated with an anti-ANXA2 monoclonal antibody (clone C-10; Santa Cruz Biotechnology, Santa Cruz, CA; Supplementary Table S7) at a final concentration of 5 or 20 μg/ml [30, 31] and then cultured for 24–96 h.

### Gene expression profiling of ANXA2 antibody-treated GBM cells and data analysis

For microarray experiments, *in vitro* transcription, hybridization and biotin labeling of RNA from GBM cells treated with anti-ANXA2 neutralizing antibody were performed according to Affymetrix 3′IVT Express Plus protocol, after 48 h of treatment with ANXA2 antibody or a matched isotype control. GeneChip Human Genome U133 Plus 2.0 (Affymetrix, Santa Clara, CA) was used.

Microarray data (CEL files) were generated using default Affymetrix microarray analysis parameters (Command Console suite software, Affymetrix). CEL files were normalized using the robust multiarray averaging expression measure of Affy-R package (www.bioconductor.org). Shrinkage *t*-test [66] was applied to identify genes that were differently expressed along ANXA2 antibody and isotype control-treated GBM cells in four independent experiments. Local False Discovery Rate (lFDR) < 0.05 was used as multiplicity correction of *p*-value to identify gene differently expressed. A heat map was generated by R software (www.R-project.org) using Euclidean distance as a distance measure between genes and Ward method for clustering probe sets. Expression data have been deposited into the Gene Expression Omnibus (GEO) database under Series Accession Number GSE76786 and are accessible without restrictions.

Gene Set Enrichment Analysis (GSEA) was performed using GSEAv2.0 with probe sets ranked by signal-to-noise ratio and statistical significance determined by 1000 permutations [67]. Gene sets permutations (< 7 replicates in each class) were used to enable direct comparisons between ANXA2 antibody and isotype control-treated GBM cells. Minimum gene set size was set to 15; maximum of probe sets was used to collapse multiple probe sets into gene. For GSEA an FDR cutoff < 0.05 was used. MgSigDataBase derived from c2 curated dataset and c5 Gene Ontology dataset were selected to obtain the enrichment gene sets.

Enrichment map was generated using Enrichment Map Cytoscape v3.2.1 plug-in [68]. Only Gene sets with FDR *q* value ≤ 0.05, derived from c5 Gene Ontolgy MSigDB GSEA were used to build the network. Node represents the functional gene sets and the size is proportional to size of gene set. Edge represents the degree of gene overlap that exist between two gene sets and the thickness is proportional to the overlap between the gene sets. To generate the gene sets relationship we used Overlap Coefficient parameters (Overlap Coefficient = [size of (A intersect B)] / [size of (minimum(A, B))], where A and B are two gene sets). Redundant gene sets with common biological function were grouped in cluster and manually labeled with Gene Ontology terms. Blue indicate enrichment in ANXA2 antibody-treated GBM cells.

For subtype classification we performed clustering analysis according to the 840 gene Verhaak classifier [29].

### Scratch-migration and invasion assays

To evaluate the effects of ANXA2 modulation on the migratory properties of GBM cells, they were cultured on 35-mm culture dishes until they reached at least 80% confluence. 24 h after antibody treatment or 48 h after ANXA2 silencing/over-expression, GBM cell monolayer has been gently scratched horizontally and vertically. After being scratched, GBM cells were washed twice with culture medium to remove cell debris and incubated until pre-determined endpoints. Migrated cells were defined as cells that moved into the scratch and detached away from the cell monolayer. Cell migration was evaluated by measuring the distance between the two edges of the scratch in at least 8 random fields by using Adobe Photoshop CS6 (Adobe Systems Incorporated, La Jolla, CA; www.adobe.com). Images were acquired by using with a Nikon TS100 inverted microscope (Nikon, Melville, NY).

To assess the invasive capacity of GBM cells depending on ANXA2 levels, soluble extracellular matrix Matrigel was dispensed in 24-well plates and allowed to gel for about 45 min at 37°C. GBM cells were then added onto Matrigel layer in a volume of 0.5ml, treated with anti-ANXA2 antibody for 72 h or 7 days at 5 or 20 μg/ ml. Images were captured with a Nikon TS100 inverted microscope (Nikon, Melville, NY). Calculation of number and length of branches and invading cells was performed with Angiogenesis plug-in from ImageJ software (https://imagej.nih.gov). Moreover, GBM invasion was also evaluated using the CultreCoat® 24 Well BME Cell Invasion Assay (Trevigen, Gaithersburg, MD) according to the manufacturer's instructions. Invasion was measured 48 h after plating, at 485–520 nm using a VICTOR spectrophotometer (Perkin Elmer, Milan, Italy).

### Chick embryo chorioallantoic membrane invasion assay

Plastic rings were placed on the chorioallantoic membrane (CAM) of fertilized White Leghorn chicken eggs at day 8 (8–10 CAMs/experimental group). Then, 6 μl of a cell suspension containing 1.5 × 10^5^ EGFP-GBM cells (HuTuP13) were injected inside the ring. CAMs were treated with a solution containing 2 μg of isotype or anti-ANXA2 antibodies every day for 3 consecutive days. On day 11, CAMs were fixed with PFA 3%, washed and mounted with Vectashield Antifade Mounting Medium (Vector Laboratories Inc. Burlingame, CA). Samples were acquired under an Axiovert 200 fluorescence microscope equipped with a EC Plan Neofluar 20x/0.5 NA objective and ApoTome system (Carl Zeiss, Oberkochen, Germany). EGFP-positive areas were quantified using Image-Pro Plus software (Media Cybernetics, Inc., Rockville, MD).

### Correlation of ANXA2^down^ signature to clinical outcome

The ANXA2^down^ gene signature was generated by using the most significant down-regulated genes after treatment of GBM cells with the monoclonal antibody against ANXA2 (fold change ≤ 0.8; Supplementary Table S5). Then, we evaluated the prognostic potential of this signature in TCGA [26], GSE13041 [28], GSE17536 [34], E-MTAB-365 [35] and GSE31210 [36] datasets. The log_2_ expression values for each sample in each dataset were centered to zero mean. The sum of the mean-centered log_2_ expression values of the ANXA2^down^ probe sets was used as the Risk Score for each subject and the 519 subjects from TCGA, 191 from GSE13041, 177 from GSE17536, 409 from E-MTAB-365 and 217 from GSE31210 were split into high- and low-risk groups greater and less than the median risk score respectively [69]. These risk groups were assessed for prognostication of OS and PFS in univariate Cox analysis (log-rank test).

### Statistics

Graphs and associated statistical analyses were generated using Graph Pad Prism 6.07 (GraphPad, La Jolla, CA). All data in bar graphs are presented as mean ± standard error of the mean (S.E.M.). Statistical significance was measured by one-way ANOVA with Newman–Keuls multiple comparison post test (for more than two comparisons) and paired *t*-test (comparison of two groups); *p < 0.05, **p < 0.01, ***p < 0.001, ****p < 0.0001. For all graphs, asterisks over brackets indicate a significant difference with another variable as indicated and asterisks over bars indicate a significant difference with the control group.

Survival analyses were performed by generating Kaplan Meier survival curves and significance calculated by log-rank (Mantel-Cox) test. In all comparisons of survival, Hazard Ratio have been calculated as ANXA2 High risk/ANXA2 Very Low risk.

Independent prognostic value of GBM patient subgroups generated on the basis of ANXA2 expression was calculated by applying a multivariate Cox analysis (Wald test) with SPSS 13 software (SPSS Inc., Chicago, IL).

## SUPPLEMENTARY MATERIALS








