# Supplementary material for: Annexin 2A sustains glioblastoma cell dissemination and proliferation
Source: Oncotarget. 2016 Jul 13;7(34):54632–49. doi: 10.18632/oncotarget.10565 (PMC5342369; doi:10.18632/oncotarget.10565)
Supplement: Supplementary file 1 [file oncotarget-07-54632-s001.pdf]

Annexin 2A sustains glioblastoma cell dissemination and proliferation

Supplementary Materials

MATERIALS AND METHODS

Immunohistochemistry

ANXA2 immunohistochemistry was conducted on 5 µm sections from 89 FFPE GBM specimens with standard procedures. Briefly, sections were re-hydrated and then antigen retrieval was performed by incubation with citrate buffer 0.01 M pH6 at 95°C. After saturation with the appropriate normal serum, slides were incubated with an anti-ANXA2 antibody (clone 5/Annexin II; BD Bioscience, San Jose, CA; Supplementary Table S7). After incubation, sections were washed and incubated with species-specific biotin-conjugated secondary antibodies (Vector Laboratories Inc. Burlingame, CA; Supplementary Table S7). ANXA2 expression was revealed by using the Dako Liquid DAB^+ Substrate Chromogen System (Dako, Glostrup, Denmark) according to manufacturer's guidelines. Tissues were counterstained with Meyer's Hematoxylin and images acquired with a Zeiss Imager M1 microscope (Carl Zeiss, Oberkochen, Germany). The specificity of each staining procedure was confirmed by replacing the primary antibodies with an Isotype control.

The expression level of ANXA2 was scored using a combined method accounting for both the staining intensity and the percentage of positive stained cells. The resulting combined score was calculated as the multiplication of the score accounting for the percentage of $ANXA2^+$ cells (0–6) and the intensity score (0–3). ANXA2 stained slides were independently evaluated by two different pathologists.

Transfection of primary GBM cells

To achieve a suitable gene silencing, GBM cells were transfected with 200pmol of two different small interfering RNAs (siRNAs) against ANXA2 as well as with a non targeting siRNA (siNEG) using the Lipofectamine RNAiMAX reagent (Thermo Fisher Scientific, Waltham, MA) according to manufacturer's instructions. siANXA2#1 siRNA sequence: (RNA)-5′-GCG ACU ACC AGA AAG CGC UGC UGU A-3′; siANXA2#2 siRNA sequence: (RNA)-5′-GAC UGA UCU GGA GAA GGA CAU UAU U-3′ (Stealth RNAi™; siANXA2#1 cat.# 10620318, siANXA2#2 cat.# 10620319).

For ANXA2-overexpression experiments, the ANXA2 complete cDNA sequence was cloned into a pcDNA3.1 empty vector in order to generate an expression vector named pcDNA3.1-ANXA2. Primers used for ANXA2 coding sequence amplification are listed in Supplementary Table S8. In over-expression experiments, GBM cells were transfected with an opportune quantity of pcDNA3.1-ANXA2 or pcDNA3.1 empty vector using the TransIT®-LT1 Transfection Reagent (Mirus Bio LLC, Madison, WI) according to manufacturer's instructions. Transfected cells were then cultured for 24–96 h depending on the experimental endpoints. The analysis of silencing or overexpression specificity was achieved by WB and qRT-PCR.

Western blot

Equal amounts of proteins (10 µg) extracted from primary GBM cells were resolved using a SDS-PAGE gels and transferred to polyvinylidene difluoride (PVDF) Immobilon-p membrane (Merk-Millipore, Darmstadt, Germany). Membranes were blocked with I-block™ (Thermo Fisher Scientific, Waltham, MA) for at least 1 hour at room temperature and then were incubated overnight at 4°C under constant shaking with the primary antibody against ANXA2 (clone 5/Annexin II; BD Bioscience, San Jose, CA; Supplementary Table S7). Membranes were next incubated with HRP-labeled goat anti-mouse IgG (1:50000; Sigma-Aldrich, Milan, Italy; Supplementary Table S7) for 60 minutes. All membranes were visualized using ECL Select (GE Healthcare, Catania, Italy) and exposed to Amersham Hyperfilm ECL (GE Healthcare). β-actin staining (Sigma-Aldrich S.r.l., Milan, Italy; Supplementary Table S7) was used as loading control. Original unmodified membrane acquisitions are reported as Supplementary Fig. S12.

Reverse transcription and real-time PCR

RNA was extracted from GBM cells using TRIzol reagent (Thermo Fisher Scientific, Waltham, MA) according to manufacturer's instructions and 1–2 µg of total RNA reverse-transcribed using SuperScript RNAse II Reverse Transcriptase (Thermo Fisher Scientific, Waltham, MA). Quantitative RT-PCR reactions were run in triplicate using Platinum SYBR Green Q-PCR

Super Mix (Thermo Fisher Scientific, Waltham, MA). Fluorescent emission was recorded in real-time (Sequence Detection System 7900HT, Applied Biosystems, Foster City, CA). The specificity of primers was confirmed for every PCR run by dissociation curve analysis. Primers used are listed in Supplementary Table S8 and their specificity was confirmed by the software Human BLAT Search (http://genome.ucsc.edu). Relative RNA quantities were normalized to *GUSB* according to the ΔΔCt Method.

Immunofluorescence

GBM cells were cultured on 4-well chamber slides (BD Bioscience, San Jose, CA), treated depending on the experimental plan. After treatment, cells were fixed in cold 4% formaldehyde and stored at +4°C prior to analysis. Primary antibody staining was performed for Nestin (Merk-Millipore, Darmstadt, Germany), Ki67, GFAP, S100 (all from Dako, Glostrup, Denmark), MAP2 (Sigma-Aldrich, Milan, Italy), OSP (Abcam, Cambridge, UK), ANXA2 (clone C-10; Santa Cruz Biotechnology, Santa Cruz, CA), FITC-coniugated Phalloidin (1:1000; Sigma-Aldrich, Milan, Italy) and Sox2 (Cell Signaling Technology). For additional details on antibodies used for immunofluorescence, please see Supplementary Table S7. After incubation, cells were washed and incubated with species-specific secondary antibodies conjugated to Alexa dyes (Thermo Fisher Scientific, Waltham, MA; Supplementary Table S7). Cells were counterstained with DAPI (1:10000; Sigma-Aldrich, Milan, Italy) to measure total cell number. Staining was visualized by epifluorescence with a ViCo microscope (Vico, Nikon, Melville, NY).

Cytofluorimetric analyses

CD133 were incubated with anti-human CD133 (clone CD133/1-PE; Miltenyi Biotec, Bergisch Gladbach, Germany) as previously described (Supplementary Table S7). Relative percentages of different subpopulations were calculated based on live gated cells (as indicated by physical parameters, side scatter and forward scatter). Unlabeled cells and cells incubated with appropriate isotype control antibodies were first acquired to ensure labelling specificity. To perform cell cycle analysis, GBM cells were collected after 72 h of treatment (anti-ANXA2 antibody, ANXA2 silencing or over-expression), centrifuged and fixed with ice-cold ethanol (70%). Cells were then treated with permeabilization buffer containing RNAse A, 0.1% Triton X-100 and propidium iodide (PI) at room temperature for 30 minutes prior to analysis. DNA histograms were analyzed using Multi- Cycle® for Windows (Phoenix Flow Systems, San Diego, CA).

Analysis of BrdU incorporation was performed with a BrdU Staining Kit for Flow Cytometry FITC (eBioscience, Hatfield, UK) according to the manufacturer's instructions. BrdU was incubated with cells for 72 h. For all stainings, samples were acquired on a Cytomic FC500 flow cytometer (Beckman Coulter, Brea, CA).

REFERENCES

1. Cancer Genome Atlas Research N. Comprehensive genomic characterization defines human glioblastoma genes and core pathways. Nature. 2008; 455:1061–1068.
2. Cancer Genome Atlas Research N, Weinstein JN, Collisson EA, Mills GB, Shaw KR, Ozenberger BA, Ellrott K, Shmulevich I, Sander C, Stuart JM. The Cancer Genome Atlas Pan-Cancer analysis project. Nat Genet. 2013; 45:1113–1120.
3. Lee Y, Scheck AC, Cloughesy TF, Lai A, Dong J, Farooqi HK, Liau LM, Horvath S, Mischel PS, Nelson SF. Gene expression analysis of glioblastomas identifies the major molecular basis for the prognostic benefit of younger age. BMC Med Genomics. 2008; 1:52.
4. Verhaak RG, Hoadley KA, Purdom E, Wang V, Qi Y, Wilkerson MD, Miller CR, Ding L, Golub T, Mesirov JP, Alexe G, Lawrence M, O'Kelly M, et al. Integrated genomic analysis identifies clinically relevant subtypes of glioblastoma characterized by abnormalities in PDGFRA, IDH1, EGFR, and NF1. Cancer Cell. 2010; 17:98–110.

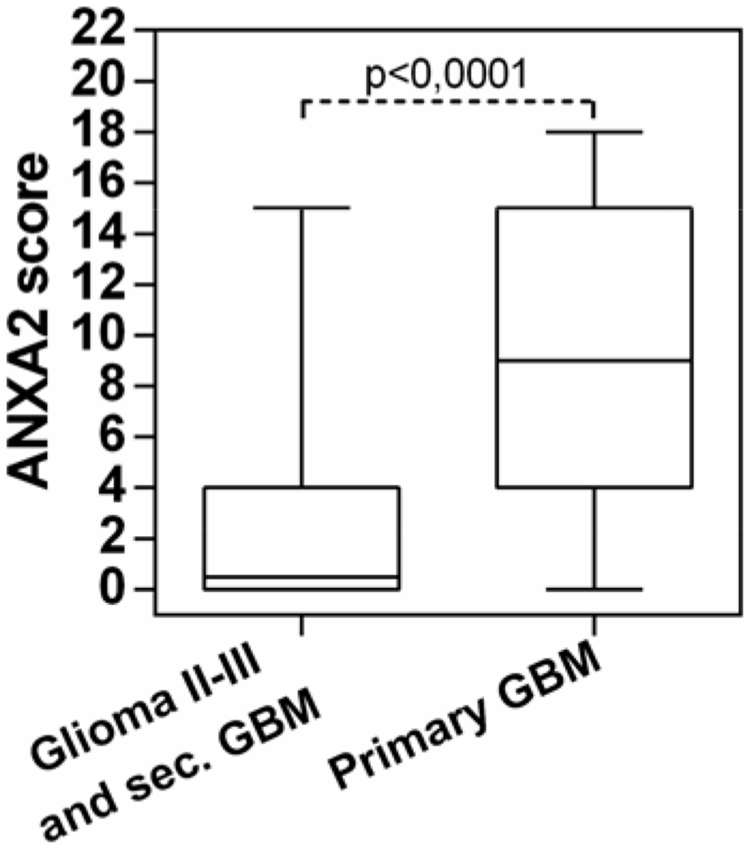

Supplementary Figure S1: ANXA2 protein expression is upregulated in GBM. Box plot showing the comparison between ANXA2 IHC scores of low grade gliomas/secondary GBMs ($N = 20$) and primary GBM tumours ($N = 69$). Significance has been calculated by Mann-Whitney test.

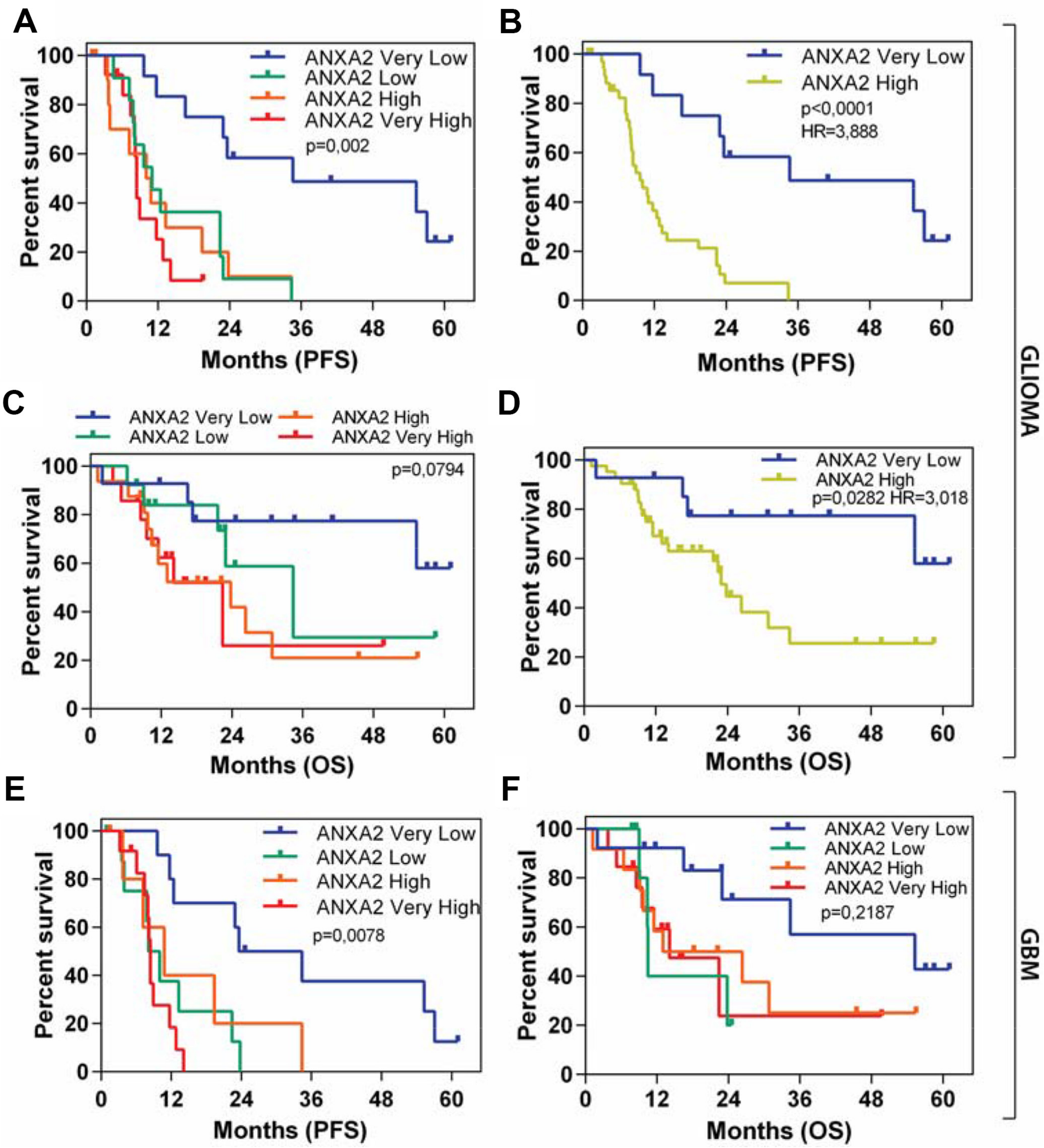

Supplementary Figure S2: ANXA2 protein expression impacts glioma patient survival. (**A–D**) Kaplan Meier curves showing PFS and OS of glioma patients divided in four subgroups (A and C) depending on ANXA2 IHC score (ANXA2 Very Low: IHC score < 25° percentile; ANXA2 Low: IHC score < 50° percentile; ANXA2 High: IHC score < 75° percentile; ANXA2 Very High: IHC score ≥ 75° percentile). Right panels reports PFS (B) and OS (D) of glioma patients divided in two subgroups depending on ANXA2 IHC score (ANXA2 Very Low: IHC score < 25° percentile; ANXA2 High: IHC score > 25° percentile). (**E** and **F**) Kaplan Meier curves showing PFS (E) and OS (F) of GBM patients divided in four quartiles on the basis of ANXA2 IHC score as previously reported.

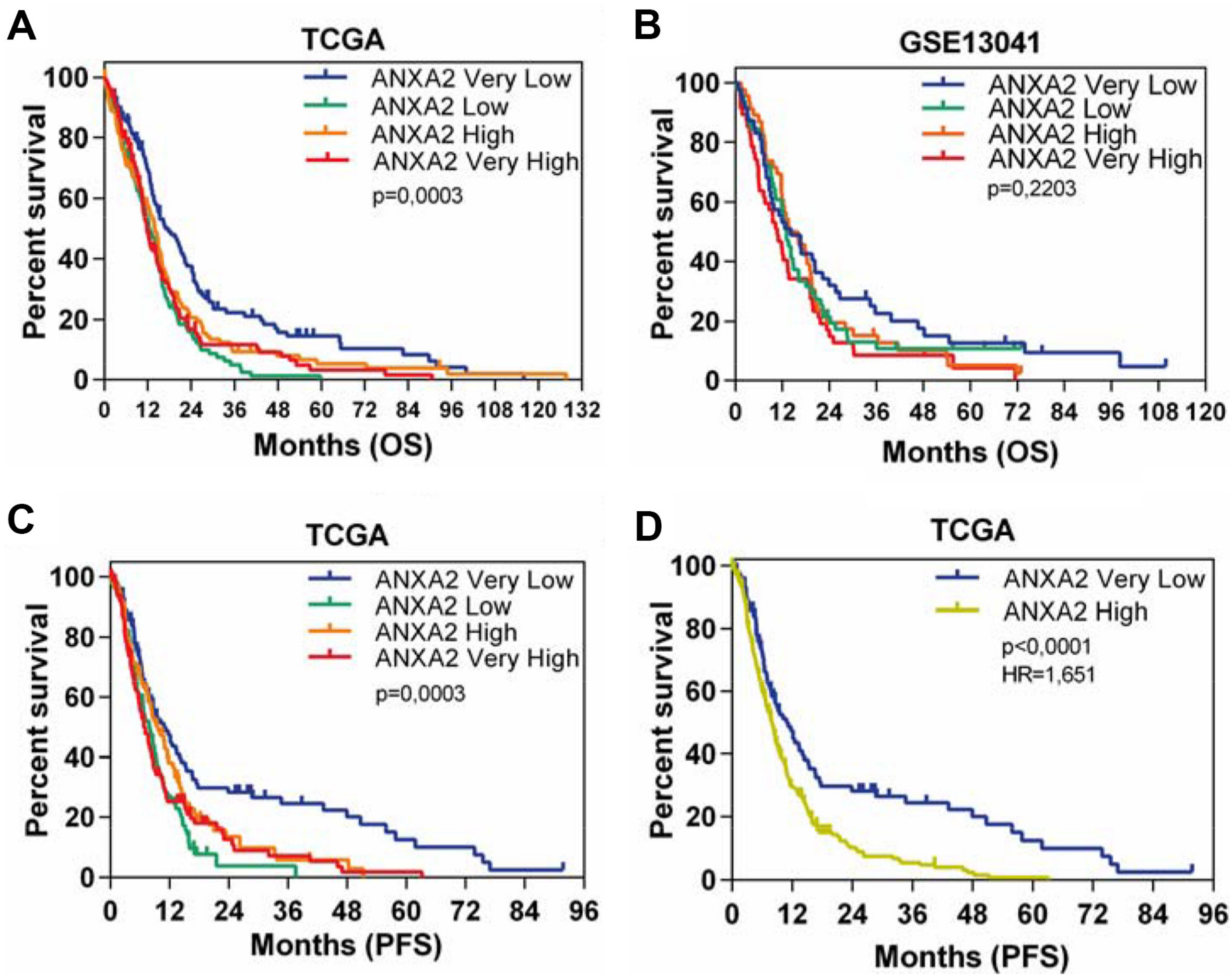

Supplementary Figure S3: ANXA2 mRNA expression impacts GBM patient survival. (**A** and **B**) Kaplan Meier curves showing OS of GBM patients retrieved from TCGA (A; *N* = 519 patients) [1, 2] or GSE13041 (B; *N* = 191 patients) [3] datasets. GBM patients have been divided in four quartiles on the basis of ANXA2 mRNA expression levels as previously described. (**C** and **D**) Kaplan Meier curves showing PFS of GBM patients from TCGA divided in four quartiles (C) or in two subgroups (D; < or ≥ 25° percentile) on the basis of ANXA2 mRNA expression levels.

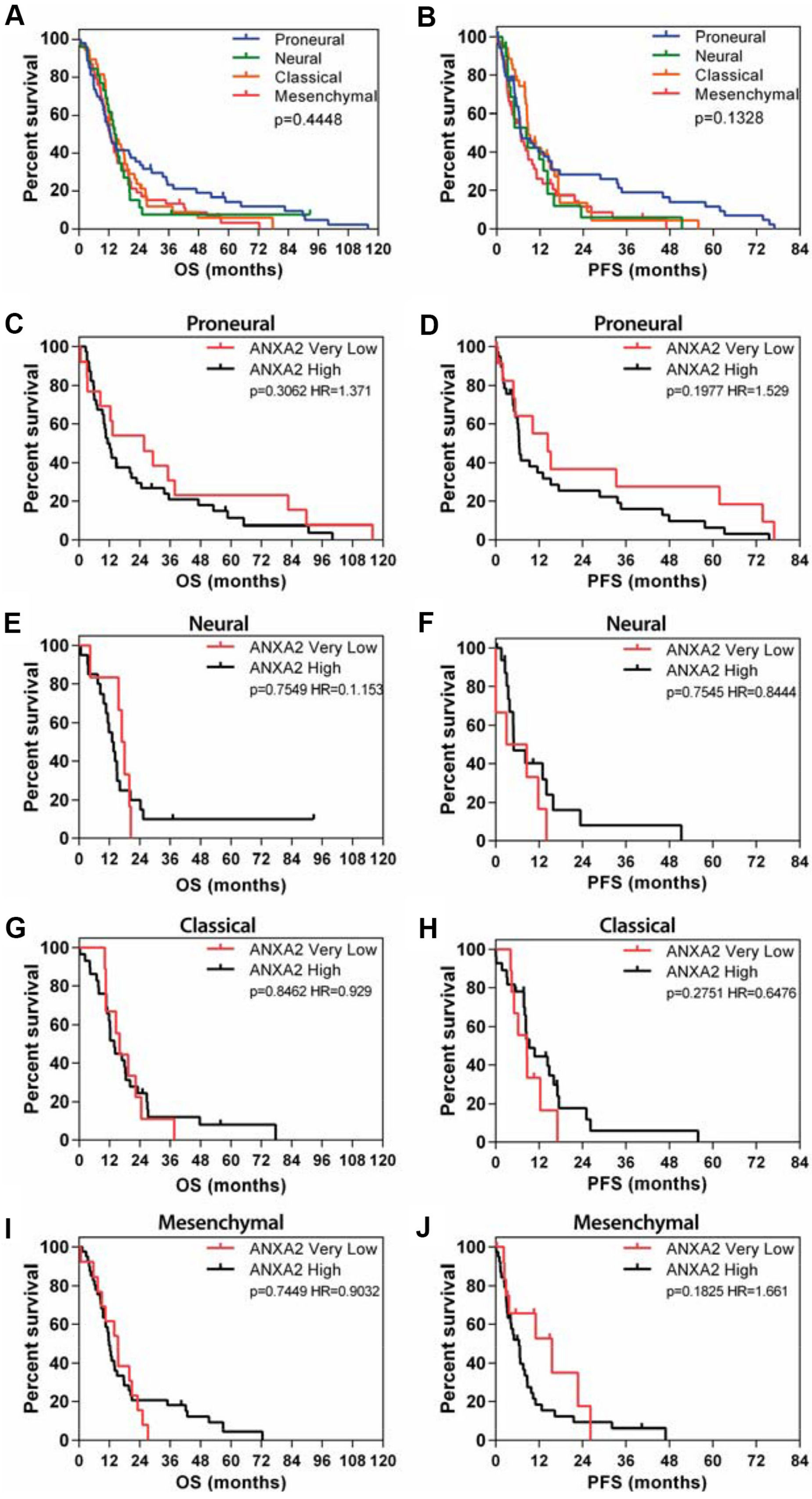

Supplementary Figure S4: ANXA2 prognostic potential is not dependent on the GBM molecular subclass. (**A** and **B**) Kaplan Meier curves showing OS (A) and PFS (B) of GBM patients retrieved from TCGA (N = 172) and classified according to the molecular subtypes by Verhaak *et al.* [4]. (**C–J**) Kaplan Meier curves reporting survival analysis (OS, left and PFS, right panels) of TCGA patients divided in two subgroups on the basis of ANXA2 expression (< or ≥ 25° percentile) in the Proneural (C and D), Neural (E and F), Classical (G and H) and Mesenchymal (I and J) molecular subtypes.

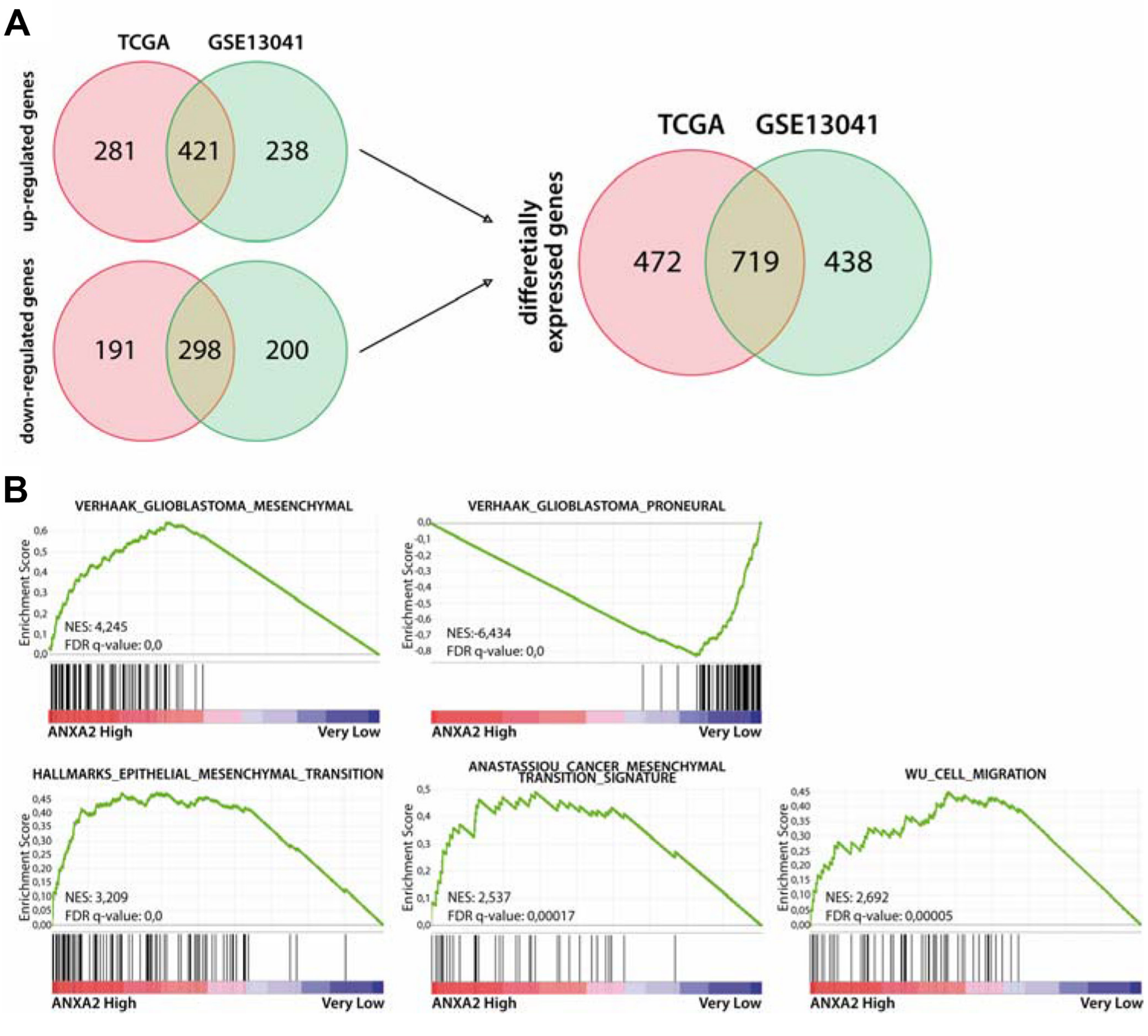

Supplementary Figure S5: GSEA analysis of differentially expressed genes in ANXA2 High vs. ANXA2 Very Low GBM patients. (**A**) Venn diagrams reporting the number of differentially expressed genes between GBM tumours expressing high levels (ANXA2 High; ≥ 25° percentile) or very low (ANXA2 Very Low; < 25° percentile) levels of ANXA2 mRNA in patients retrieved from TCGA (orange) or GSE13041 (green) datasets. (**B**) GSEA of commonly up-regulated genes in ANXA High patients from TCGA and GSE13041 datasets showing positive enrichment for genes of the Mesenchymal molecular subtype [4], EMT and cell migration, and negative enrichment for genes of the Proneural molecular subtype [4]. Plots were generated by c2 curated gene sets in the GSEA MSigDatabase. The green curves show the enrichment score and reflects the degree to which each gene (black vertical lines) is represented at the top or bottom of the ranked gene list. The heat map indicates the relative abundance (red to blue) of the genes specifically enriched in the ANXA2 High as compared with the ANXA2 Very Low GBM patients.

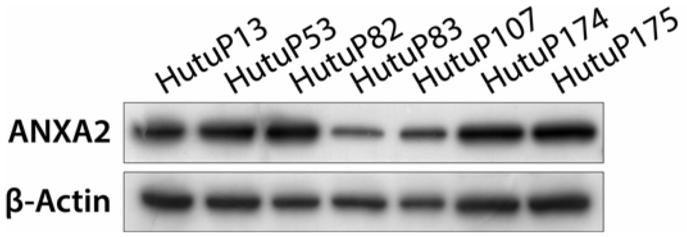

Supplementary Figure S6: ANXA2 protein expression in primary GBM cell cultures. Western Blot analysis of ANXA2 protein expression in seven primary GBM cells used in this study. β-Actin expression has been used as loading control. 10 µg of protein lysate has been loaded in each lane.

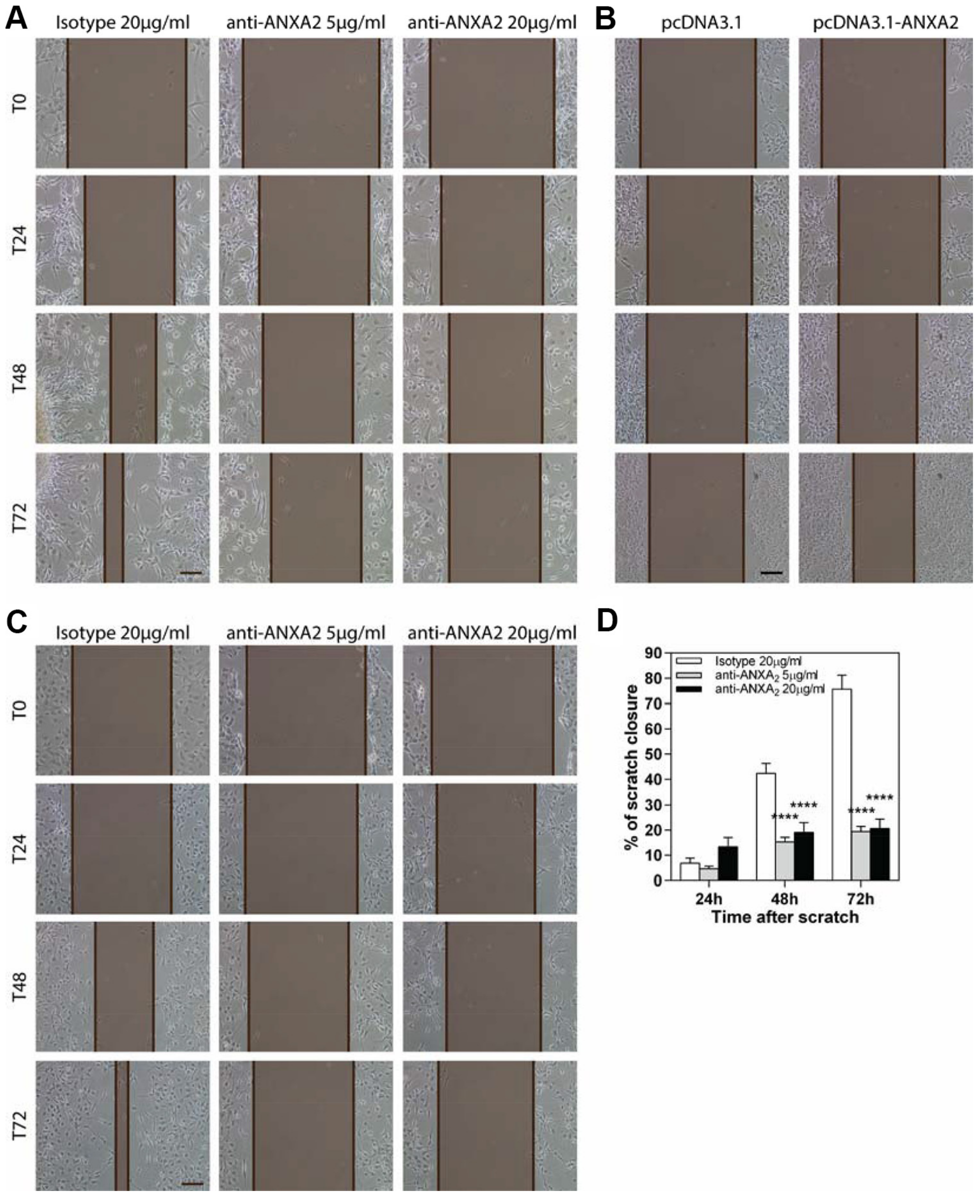

Supplementary Figure S7: Scratch assay on GBM and SVZ-derived cells at different time points. Representative images showing the ability of GBM cells to close the wound at 24, 48 and 72 h after scratching the cell monolayer during scratch assays performed on primary GBM cells treated with the monoclonal anti-ANXA2 antibody (**A**; HuTuP175) or ANXA2 over-expressing (**B**; HuTuP83) cells and SVZ-derived SC23 normal cells treated with the monoclonal anti-ANXA2 antibody (**C**) with relative quantification (N = 8). The distance between the two edges of the scratch is marked in brown and has been quantified by using Adobe Photoshop CS6. Original magnification 10×; bar:50 µm. ****$p < 0.001$ by one-way ANOVA analysis.

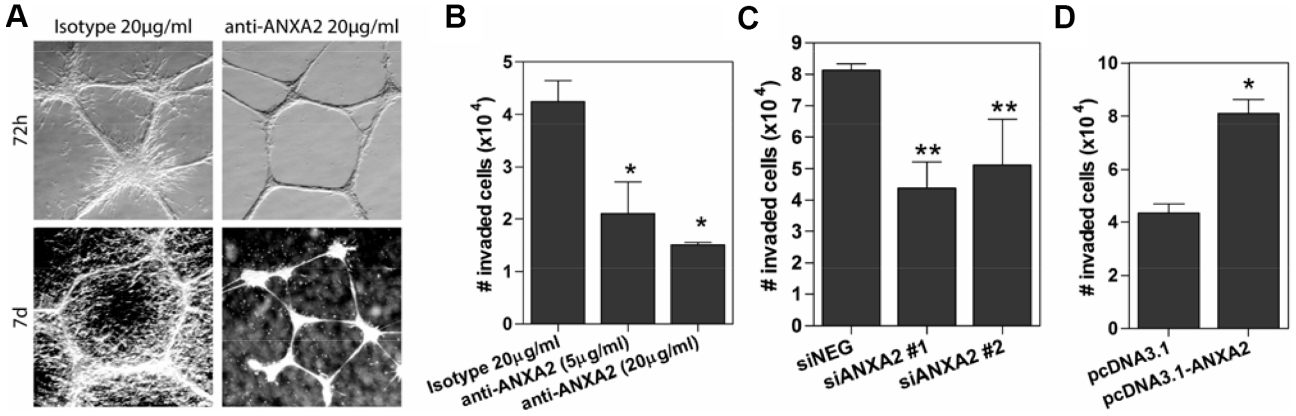

Supplementary Figure S8: Modulation of primary GBM cell invasion by ANXA2 inhibition, gene silencing or over-expression. (**A**) Representative images of primary GBM cells (HuTuP176), endowed with reticulate growth, plated on Matrigel-coated dishes and treated with anti-ANXA2 antibody for the indicated timepoints. Original magnification 4x. (**B**, **C** and **D**) Bar graphs reporting the absolute cell number of BME-invaded GBM cells upon ANXA2 inhibition (B; $N = 3$ for HuTuP13 and HuTuP175), gene silencing (C; $N = 3$ for HuTuP13 and HuTuP174) or over-expression (D; $N = 4$ for HuTuP83) as quantified by Cultrecoats® BME-based assays as described in the Methods section.

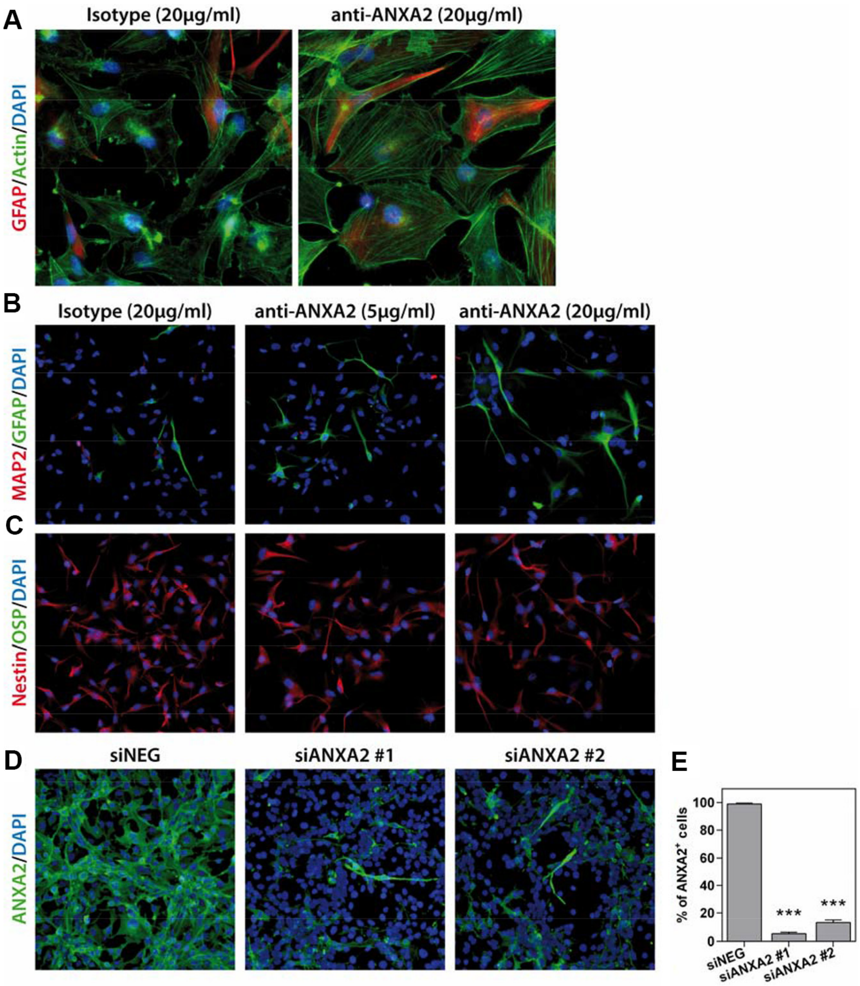

Supplementary Figure S9: Immunofluorescence analysis of ANXA2 inhibited/silenced GBM cells. (**A–C**) Representative immunofluorescence images of GBM cells treated either with isotype or ANXA2 antibody and double stained for GFAP (red)/phalloidin (green) (A), MAP2 (red)/GFAP (green) (B) and Nestin (red)/OSP (green) (C). Images show a peculiar distribution of GFAP and Actin fibers within the cell cytoplasm. (**D** and **E**) Representative immunofluorescence images showing primary GBM cells (HuTuP13) silenced for ANXA2 and stained with anti-ANXA2 antibody (green; D) and bar ghaph reporting relative quantification of $ANXA2^{+}$ cells (E). Cell nuclei have been counterstained with DAPI (blue). Original magnification 10–20x. ***$p < 0.001$ by one-way ANOVA analysis.

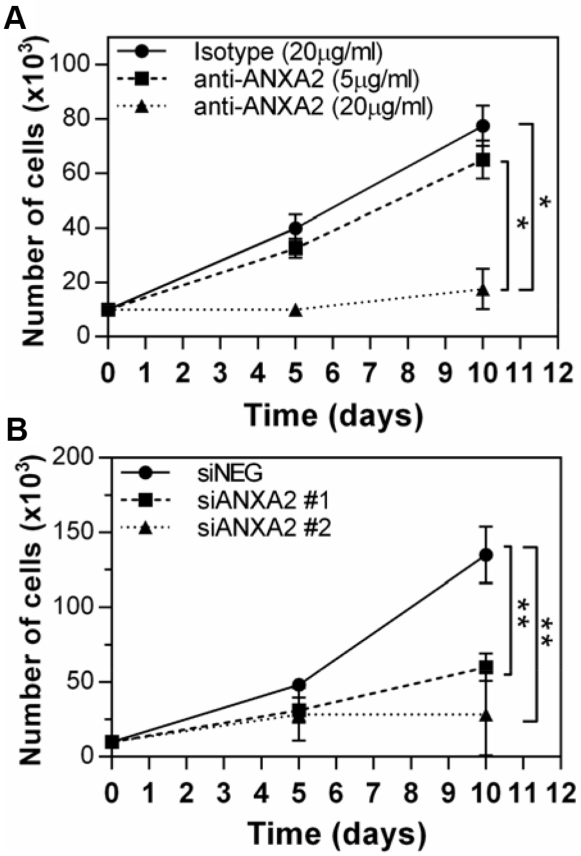

Supplementary Figure S10: Long term anti-proliferative effects of ANXA2 inhibition/silencing in GBM cells. Growth curves generated by cell count (trypan blue exclusion) at different time-points after ANXA2 inhibition (HuTuP175; A) or gene silencing (HuTuP13; B). *$p < 0.05$, **$p < 0.001$ by one-way ANOVA analysis.

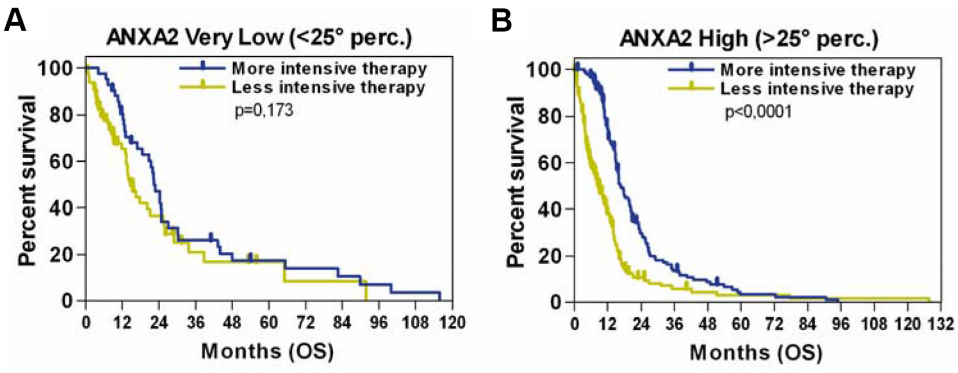

Supplementary Figure S11: Impact of aggressive treatment on GBM patients depending on ANXA2 expression. Kaplan Meier curves reporting OS of GBM patients either treated with a single cycle of mono or concurrent therapy (Less Intensive), or by additional cycles of treatment (More Intensive) depending on their transcriptional expression of ANXA2 (< or ≥ 25° percentile in (**A**) and (**B**) respectively).

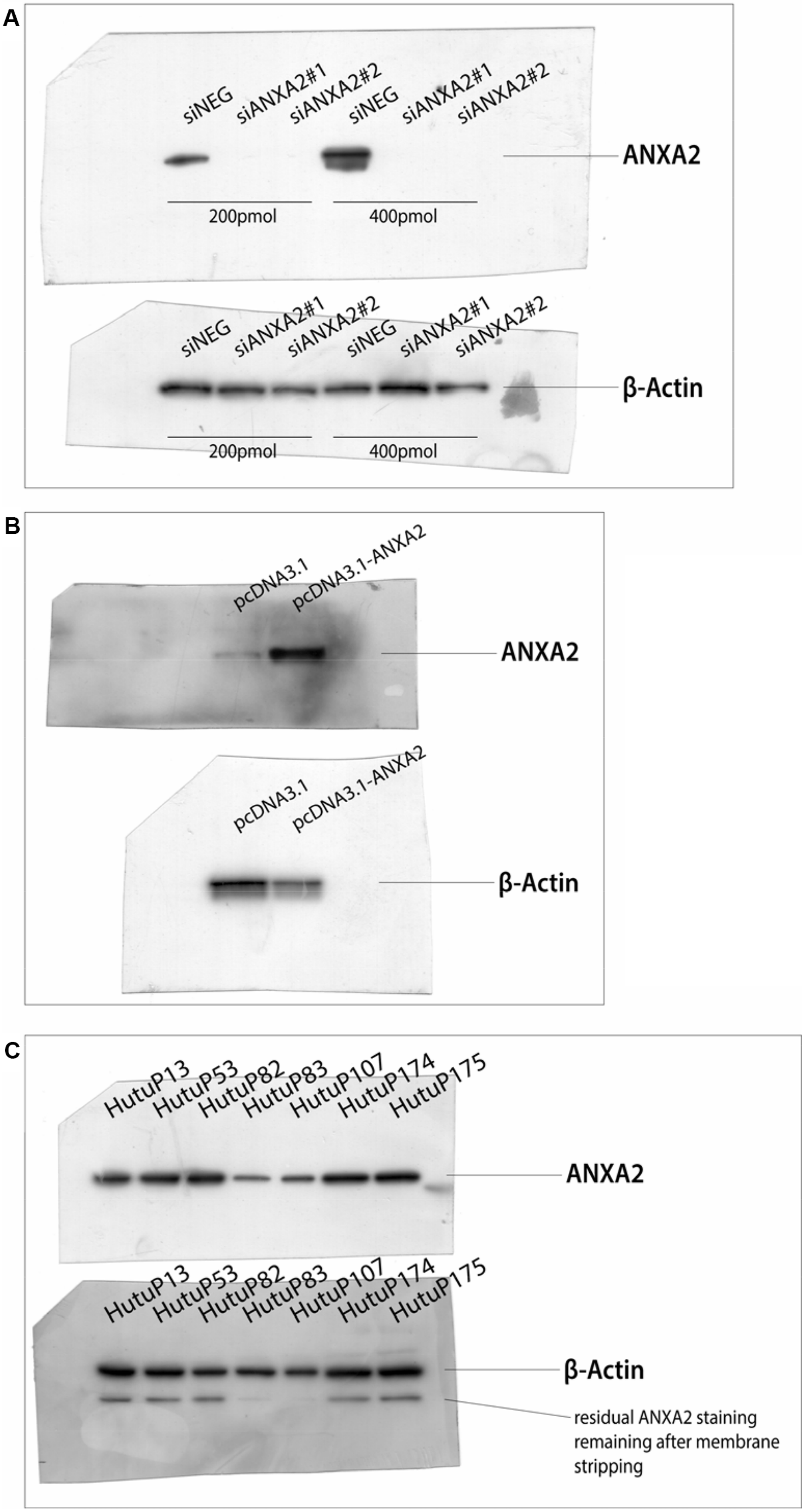

Supplementary Figure S12: Unedited acquisitions of Western Blot membranes. (**A**) Unedited acquisitions of WB showed in Figure 2D. (**B**) Unedited acquisitions of WB showed in Figure 2E. (**C**) Unedited acquisitions of WB showed in Supplementary Figure S6.

Supplementary Table S1: Newman-Keuls multiple comparisons test from GSE4290

**Comparison**	***p* value**
Normal Brain vs. Glioma II	$p < 0.05$
Normal Brain vs. Glioma III	$p < 0.001$
Normal Brain vs. GBM	$p < 0.0001$
Glioma II vs. Glioma III	$p < 0.05$
Glioma II vs. GBM	$p < 0.0001$
Glioma III vs. GBM	$p < 0.001$

Supplementary Table S2: Clinical characteristics of glioma patients included in survival and multivariate analyses. See Supplementary_Tables_S2

Supplementary Table S3: Common differentially expressed genes between TGCA and GSE13041 derived from comparison between ANXA2 Very Low and ANXA2 High patients. See Supplementary_Tables_S3

Supplementary Table S4: primary GBM cells used in the study

Code	Diagnosis	Age (y)	Sex
**HuTuP13**	GBM	67	male
**HuTuP53**	GBM	70	male
**HuTuP82**	GBM	50	male
**HuTuP83**	GBM	55	male
**HuTuP107**	GBM	65	male
**HuTuP174**	GBM	69	male
**HuTuP175**	GBM	54	female
**HuTuP176**	GBM	59	male

Supplementary Table S5: Differentially expressed genes between Isotype and anti-ANXA2 antibody treated GBM cells. See Supplementary_Tables_S5

Supplementary Table S6: Summary of Log-rank analysis for ANXA2-dependent Risk Score on TCGA, GSE13041, GSE17536, E-MTAB-365 and GSE31210 cohorts of cancer patients

Tumour type (origin of data)	Survival	ANXA2 status	Median Survival (months)	Log-rank (Mantel-Cox) p value	Hazard Ratio (logrank) Risk score low/ Risk score high
GBM from TCGA	PFS	Risk Score High<br>Risk Score Low	7.83<br>8.72	0.019	1.288
	OS	Risk Score High<br>Risk Score Low	12.93<br>14.41	0.0261	1.242
GBM from GSE13041	OS	Risk Score High<br>Risk Score Low	11.7<br>13.55	0.0486	1.353
Colon cancer from GSE17536	DSS	Risk Score High<br>Risk Score Low	77.6<br>N.D.	0.006	2.149
Breast cancer from E-MTAB-365	RFS	Risk Score High<br>Risk Score Low	N.D.<br>N.D.	0.002	1.739
Lung adenocarcinoma from GSE31210	RFS	Risk Score High<br>Risk Score Low	N.D.<br>N.D.	0.0605	1.61

N.D. Not Determined.

Supplementary Table S7: antibodies used in this study

Antibody (Clone)	Host	Use	Dilution	Producer	Catalog#
Annexin II (5/Annexin II)	mouse	IHC	1:2000	BD Bioscience	610068
Annexin II (C-10)	mouse	IF<br>*In vitro* neutralization<br>*In vivo* neutralization	1:100<br>5-20μg/ml<br>2μg/egg	Santa Cruz Biotechnology	sc-28385
β-actin (AC-74)	mouse	WB	1:20000	Sigma-Aldrich	A5316
CD133-PE (AC133/1)	mouse	FC	1:25	Miltenyi Biotec	130-080-801
GFAP	rabbit	IF	1:1000	Dako	Z0334
Ki67 (MIB-1)	mouse	IF	1:100	Dako	M7240
MAP2 (AP-20)	mouse	IF	1:100	Sigma-Aldrich	M1406
Nestin (10C2)	mouse	IF	1:200	Millipore	MAB5326
OSP	rabbit	IF	1:200	Abcam	ab53041
S100	rabbit	IF	1:400	Dako	Z0311
Sox2 (D6D9)	rabbit	IF	1:200	Cell Signaling Technology	3579
anti-mouse-Alexa594	goat	IF	1:1000	Life Technologies	A11005
anti-mouse-Alexa488	goat	IF	1:1000	Life Technologies	A11001
anti-rabbit-Alexa488	goat	IF	1:1000	Life Technologies	A11008
Anti-rabbit-Alexa594	goat	IF	1:1000	Life Technologies	A11012
anti-mouse-HRP	goat	WB	1:50000	Perkin Elmer	NEF822001EA

IHC: Immunohistochemistry; IF: immunofluorescence; WB: western blot; FC: flow cytometry.

Supplementary Table S8: Sequence of primers used in this study

Gene	Sequence (5′-3′)
ANXA2 forward	GGGGACGCGAGATAAGGTCC
ANXA2 reverse	CGCTTTCTGGTAGTCGCCCT
ANXA2_CDS forward	ATGGGCCGCCAGCTAGCG
ANXA2_CDS reverse	TCAGTCATCTCCACCACACAGGTAC
ANXA2_BamHI-5′	ACTGGGATCCATGGGCCGCCAGCTAGCG
ANXA2_XhoI-3′	ACTGCTCGAGTCAGTCATCTCCACCACACAGGTAC
ADAM12 forward	CATCGGCATGGCCCCAATCA
ADAM12 reverse	GGCTGCACCAAGGGGATTGT
COL5A1 forward	CCCTGACAAGAAGTCCGAAGGG
COL5A1 reverse	GCAGCCGCAGGAAGGTCAT
DNAH9 forward	GGCCCGACCGGATGACCTAT
DNAH9 reverse	TCCACCCCTGGAGACAGGATG
FBN1 forward	GCTGCGAGTGTCCCTTTGGT
FBN1 reverse	GGGCTCAAATCCCTCCTCGC
HMMR forward	GGAGTGCCAGTCACCTTCAGT
HMMR reverse	ACATCATAAGCACCTGGAGATGG
PLAT forward	GCCCGATTCAGAAGAGGAGCC
PLAT reverse	ACTGTGCCCTGCCACTGTTG
SDK2 forward	CCGTCAACGACGTGGGGAAA
SDK2 reverse	ACTGGTTGGTTCGACCGCTG
SEMA5A forward	CCCAGGCGCTGGATGACTG
SEMA5A reverse	GTGTGGAAAGTGCCAAGGAGAGAG
COCH forward	AAGGGAGAGCCCCAACAAGAAC
COCH reverse	AGGTCATCCAGAGGTGCCCAA
MYO1B forward	GGGTGGAAATGCCGCACACA
MYO1B reverse	CGAGCCTTCCAACCCCGGATA
MYL5 forward	GTACCGACGCCGAGGAGACC
MYL5 reverse	CGATGGAGGCGAACTGGAACA
CCNA2 forward	CGTGAAGATGCCCTGGCTTTTA
CCNA2 reverse	TGGTACTTCATTAACACTCACTGGC
MKI67 forward	GCCTGCTCGACCCTACAGAG
MKI67 reverse	ACTGCGGTTGCTCCTTCACTG
CDCA3 forward	GGACTCAGATCCCCGCTCTC
CDCA3 reverse	GGTACCCAGAGGCAAGTCCAA
CDK1 forward	AAGCCGGGATCTACCATACCC
CDK1 reverse	GCAGTACTAGGAACCCCTTCCTCT
KIF14 forward	CCTCTGCAGGAAAAGACCCCT
KIF14 reverse	CGTGTCAGGGTGTTCCACAGTT
KIF20A forward	GAACGAACGCAGCGCGTAAT
KIF20A reverse	GCCTAGGTCCGAAGACGTGC
GUSB forward	GAAAATACGTGGTTGGAGAGCTCATT
GUSB reverse	CCGAGTGAAGATCCCCTTTTTA
