# Supplementary material for: Annexin 2A sustains glioblastoma cell dissemination and proliferation
Source: Oncotarget. 2016 Jul 13;7(34):54632–49. doi: 10.18632/oncotarget.10565 (PMC5342369; doi:10.18632/oncotarget.10565)
Supplement: Supplementary file 4 [file oncotarget-07-54632-s004.docx]

**Supplementary Table S2: Clinical characteristics of glioma patients included in survival and multivariate analyses**

| **Sample ID** | **Age (y)** | **Gender** | **Grade** | **Occurence** | **Site of Lesion** | **Performance Score** | **Methylation of MGMT promoter** | **IDH mutation** | **Surgery** |
| --- | --- | --- | --- | --- | --- | --- | --- | --- | --- |
| **HuTuP07** | 66 | m | 4 | primary | frontal | 1 | u | wt | GTR |
| **HuTuP10** | 75 | f | 4 | primary | temporal | n/a | n/a | n/a | GTR |
| **HuTuP14** | 39 | f | 4 | primary | temporal | 0 | u | wt | GTR |
| **HuTuP16** | 66 | m | 4 | primary | parietal | n/a | n/a | n/a | GTR |
| **HuTuP17** | 65 | f | 3 | primary | parietal | 1 | m | mut | GTR |
| **HuTuP20** | 60 | m | 4 | primary | frontal | 1 | m | wt | GTR |
| **HuTuP26** | 62 | m | 4 | primary | temporal | n/a | n/a | n/a | GTR |
| **HuTuP31** | 53 | m | 4 | primary | frontal | 0 | m | mut | GTR |
| **HuTuP34** | 57 | f | 4 | primary | occipital | 1 | u | wt | GTR |
| **HuTuP36** | 49 | f | 4 | primary | temporal | n/a | n/a | n/a | GTR |
| **HuTuP37** | 79 | f | 4 | primary | temporal | 0 | m | wt | GTR |
| **HuTuP40** | 68 | f | 4 | primary | parietal | n/a | n/a | n/a | STR |
| **HuTuP47** | 81 | f | 4 | primary | frontal | n/a | n/a | n/a | GTR |
| **HuTuP53** | 70 | m | 4 | primary | parietal | n/a | n/a | n/a | GTR |
| **HuTuP55** | 62 | m | 4 | primary | frontal | 1 | m | wt | GTR |
| **HuTuP56** | 50 | m | 4 | primary | frontal | 1 | u | wt | GTR |
| **HuTuP58** | 63 | f | 4 | primary | temporal | 3 | u | wt | GTR |
| **HuTuP60** | 62 | m | 4 | primary | parietal | 2 | m | wt | STR |
| **HuTuP63** | 40 | f | 4 | secondary | parietal | 0 | u | mut | GTR |
| **HuTuP65** | 56 | f | 4 | secondary | temporal | 0 | m | mut | GTR |
| **HuTuP67** | 52 | m | 4 | primary | occipital | 1 | u | wt | GTR |
| **HuTuP69** | 47 | m | 4 | secondary | frontal | 1 | u | wt | GTR |
| **HuTuP70** | 43 | m | 4 | primary | frontal | 2 | u | wt | GTR |
| **HuTuP77** | 62 | m | 2 | primary | occipital | 1 | m | mut | GTR |
| **HuTuP82** | 53 | m | 4 | primary | frontal | 0 | m | wt | GTR |
| **HuTuP83** | 57 | m | 4 | primary | frontal | 2 | u | wt | GTR |
| **HuTuP88** | 71 | m | 4 | primary | frontal | 2 | u | wt | GTR |
| **HuTuP89** | 47 | f | 3 | primary | parietal | 0 | m | mut | GTR |
| **HuTuP91** | 38 | f | 2 | primary | parietal | 2 | m | wt | GTR |
| **HuTuP95** | 68 | m | 4 | primary | frontal | 1 | m | wt | GTR |
| **HuTuP97** | 52 | m | 2 | primary | frontal | 1 | u | wt | GTR |
| **HuTuP99** | 42 | m | 4 | primary | temporal | 3 | n/a | n/a | GTR |
| **HutuP100** | 59 | m | 4 | primary | temporal | 0 | n/a | n/a | STR |
| **HuTuP102** | 42 | f | 2 | primary | temporal | 0 | m | mut | GTR |
| **HuTuP102** | 42 | f | 4 | primary | temporal | 0 | m | mut | GTR |
| **HuTuP106** | 74 | m | 4 | primary | occipital | 0 | m | mut | GTR |
| **HuTuP107** | 67 | m | 4 | primary | parietal | 2 | u | wt | GTR |
| **HuTuP108** | 64 | m | 4 | primary | frontal | 0 | u | wt | GTR |
| **HuTuP109** | 60 | m | 4 | primary | frontal | 1 | u | wt | GTR |
| **HuTuP113** | 50 | m | 4 | secondary | parietal | 1 | u | wt | GTR |
| **HuTuP116** | 65 | m | 4 | primary | occipital | 1 | u | wt | STR |
| **HuTuP117** | 40 | m | 4 | secondary | frontal | n/a | n/a | n/a | GTR |
| **HuTuP119** | 56 | m | 4 | primary | frontal | 3 | u | wt | GTR |
| **HuTuP120** | 56 | m | 4 | primary | temporal | 2 | u | wt | GTR |
| **HuTuP121** | 53 | f | 4 | primary | occipital | 0 | m | wt | GTR |
| **HuTuP122** | 44 | f | 4 | primary | frontal | 0 | m | mut | GTR |
| **HuTuP127** | 75 | m | 4 | secondary | occipital | 2 | m | wt | GTR |
| **HuTuP129** | 68 | m | 4 | primary | parietal | 0 | m | wt | STR |
| **HuTuP135** | 50 | f | 4 | primary | parietal | 0 | u | wt | GTR |
| **HuTuP136** | 70 | f | 4 | primary | parietal | 3 | u | wt | GTR |
| **HuTuP138** | 71 | m | 4 | primary | temporal | 1 | m | wt | GTR |
| **HuTuP142** | 65 | m | 4 | primary | temporal | 3 | u | wt | GTR |
| **HuTuP145** | 51 | m | 4 | primary | parietal | 0 | u | wt | GTR |
| **HuTuP147** | 65 | m | 4 | primary | parietal | 2 | u | wt | GTR |
| **HuTuP151** | 52 | f | 4 | primary | frontal | n/a | n/a | n/a | GTR |
| **HuTuP152** | 67 | m | 4 | primary | frontal | 1 | u | wt | GTR |
| **HuTuP153** | 79 | f | 4 | primary | temporal | 2 | n/a | n/a | GTR |
| **HuTuP154** | 68 | f | 4 | primary | temporal | 1 | u | wt | GTR |
| **HuTuP155** | 49 | f | 4 | primary | temporal | 1 | u | wt | GTR |

(y): years; m: male, f: female; u: unmethylated, m: methylated; wt: wild type, mut: mutated, GTR: gross total removal (> 90%), STR: sub-total removal (< 90%). All patients underwent standard Stupp protocol of treatment.
